# Phosphorylation-Coupled Proteolysis of the Transcription Factor MYC2 Is Important for Jasmonate-Signaled Plant Immunity

**DOI:** 10.1371/journal.pgen.1003422

**Published:** 2013-04-04

**Authors:** Qingzhe Zhai, Liuhua Yan, Dan Tan, Rong Chen, Jiaqiang Sun, Liyan Gao, Meng-Qiu Dong, Yingchun Wang, Chuanyou Li

**Affiliations:** 1 State Key Laboratory of Plant Genomics, National Centre for Plant Gene Research–Beijing, Institute of Genetics and Developmental Biology, Chinese Academy of Sciences, Beijing, China; 2 National Institute of Biological Sciences–Beijing, Zhongguancun Life Science Park, Beijing, China; 3 State Key Laboratory of Molecular Developmental Biology, Institute of Genetics and Developmental Biology, Chinese Academy of Sciences, Beijing, China; National University of Singapore and Temasek Life Sciences Laboratory, Singapore

## Abstract

As a master regulator of jasmonic acid (JA)–signaled plant immune responses, the basic helix-loop-helix (bHLH) Leu zipper transcription factor MYC2 differentially regulates different subsets of JA–responsive genes through distinct mechanisms. However, how MYC2 itself is regulated at the protein level remains unknown. Here, we show that proteolysis of MYC2 plays a positive role in regulating the transcription of its target genes. We discovered a 12-amino-acid element in the transcription activation domain (TAD) of MYC2 that is required for both the proteolysis and the transcriptional activity of MYC2. Interestingly, MYC2 phosphorylation at residue Thr328, which facilitates its turnover, is also required for the MYC2 function to regulate gene transcription. Together, these results reveal that phosphorylation-coupled turnover of MYC2 stimulates its transcription activity. Our results exemplify that, as with animals, plants employ an “activation by destruction” mechanism to fine-tune their transcriptome to adapt to their ever-changing environment.

## Author Summary

The plant hormone jasmonic acid (JA) regulates a wide range of plant immune responses involving genome-wide transcriptional reprogramming that are regulated by the basic helix-loop-helix (bHLH) Leu zipper transcription factor MYC2. As a master regulator of JA signaling, MYC2 differentially regulates the transcription of different branches of JA–responsive genes through distinct molecular mechanisms. Here, we provide evidence that phosphorylation-dependent turnover of MYC2 is coupled with its function. We show that, during JA response, high accumulation of the MYC2 protein correlates with peaked expression of early wound-responsive genes that are positively regulated by MYC2, whereas low accumulation of the MYC2 protein correlates with peaked expression of late pathogen-responsive genes that are negatively regulated by MYC2. We discovered a 12-amino-acid element in the transcription activation domain of MYC2 that is required for both the proteolysis and the transcriptional activity of MYC2. Interestingly, MYC2 phosphorylation at residue Thr328, which facilitates its turnover, is also important for the MYC2 function to regulate transcription. Together, these results reveal that phosphorylation and turnover of MYC2 are tightly linked with its function to regulate the transcription of JA–responsive genes. Our results exemplify that plants employ proteolysis-coupled transcription as mechanism to fine-tune their responses to versatile stresses.

## Introduction

Plants are continuously challenged by various biotic and abiotic stresses with diverse modes of attack. In response to an attack, plant cells undergo dramatic transcriptional reprogramming to efficiently coordinate the activation of attacker-specific immune responses so that the optimal resistance is attained. Equally importantly, when the attacking alarm is relieved, plants cells must effectively suppress their immune responses at the right time to minimize the cost of defense. Therefore, plant cells have involved elaborate regulatory mechanisms to keep defense-related gene transcription tightly in check.

Among the best-characterized molecular signals regulating plant immune responses is the jasmonic acid (JA) family of oxylipins, which orchestrate genome-wide transcriptional reprogramming of plant cells to coordinate defense-related processes. Much of our understanding of the JA signal transduction pathway has come from the recent elucidation of the molecular details of JA-regulated gene transcription through MYC2, a basic helix-loop-helix (bHLH)-type transcription factor that regulates diverse aspects of JA responses [1]–[4]. At low JA levels, the transcriptional activity of MYC2 is repressed by JASMONATE ZIM DOMAIN (JAZ) proteins, which recruit TOPLESS (TPL) to form a transcriptional repressor complex through the adaptor protein NOVEL INTERACTOR OF JAZ (NINJA) [5]–[7]. A battery of stresses, including mechanical wounding, insect attack and pathogen infection, triggers a rapid increase of cellular JA levels. Synthesized JA is conjugated with isoleucine to form the active hormone JA-Ile, which is perceived by its receptor CORONATINE INSENSITIVE1 (COI1), an F-box protein that forms an E3 ubiquitin ligase [6], [8]–[11]. JA-Ile acts as a “molecular glue” to stimulate the interaction between COI1 and JAZs, which bring JAZs for degradation and therefore relieves their repression effect on MYC2 [5], [6]. Two MYC2-like bHLH-type transcription factors, MYC3 and MYC4, were also able to interact with JAZs and act additively with MYC2 in the regulation of JA-signaled immune responses [12]. Although MYC2-mediated transcriptional regulation plays a central role in different aspects of JA-mediated immunity, how MYC2 itself is regulated at the protein level remains elusive.

In mammalian, phosphorylation and the ubiquitin-proteasome system (UPS)-mediated proteolysis are prominent posttranslational mechanisms that control transcription factors [13]. One of the most extensively studied transcription factors whose activity is under the control of UPS-mediated proteolysis is the Myc oncoprotein, which is a bHLH-type transcription factor that shares structural features with MYC2 [14]–[16]. Myc is highly unstable and, surprisingly, the Myc degron–region that signals Myc destruction, is closely overlapped with its transcriptional activation domain (TAD) [17], [18], revealing a functional connection between protein destruction and gene activation. A growing body of evidence demonstrates that this functional overlap between degrons and TADs is not unique to Myc, but reflects a general phenomenon for most unstable transcription factors, leading to the “activation by destruction” hypothesis, in which the UPS-mediated turnover of transcription factors is essential for their ability to activate gene transcription [13], [19], [20]. Whereas “activation by destruction” is a general phenomenon in the mammalian system, evidence that this paradigm is also involved in the regulation of transcription factors in the plant kingdom is lacking.

In this report, we investigated if MYC2 is regulated by posttranscriptional mechanisms. Our findings revealed that UPS-mediated proteolysis is involved directly and mechanistically in the regulation of MYC2 and demonstrate that plants employ proteolysis-coupled transcription as a mechanism to control their responses to various environmental stresses.

## Results

### Temporal Correlation of MYC2 Protein Accumulation with Its Differential Effects on the Transcription of Wound- and Pathogen-Responsive Genes

**Figure 1:**
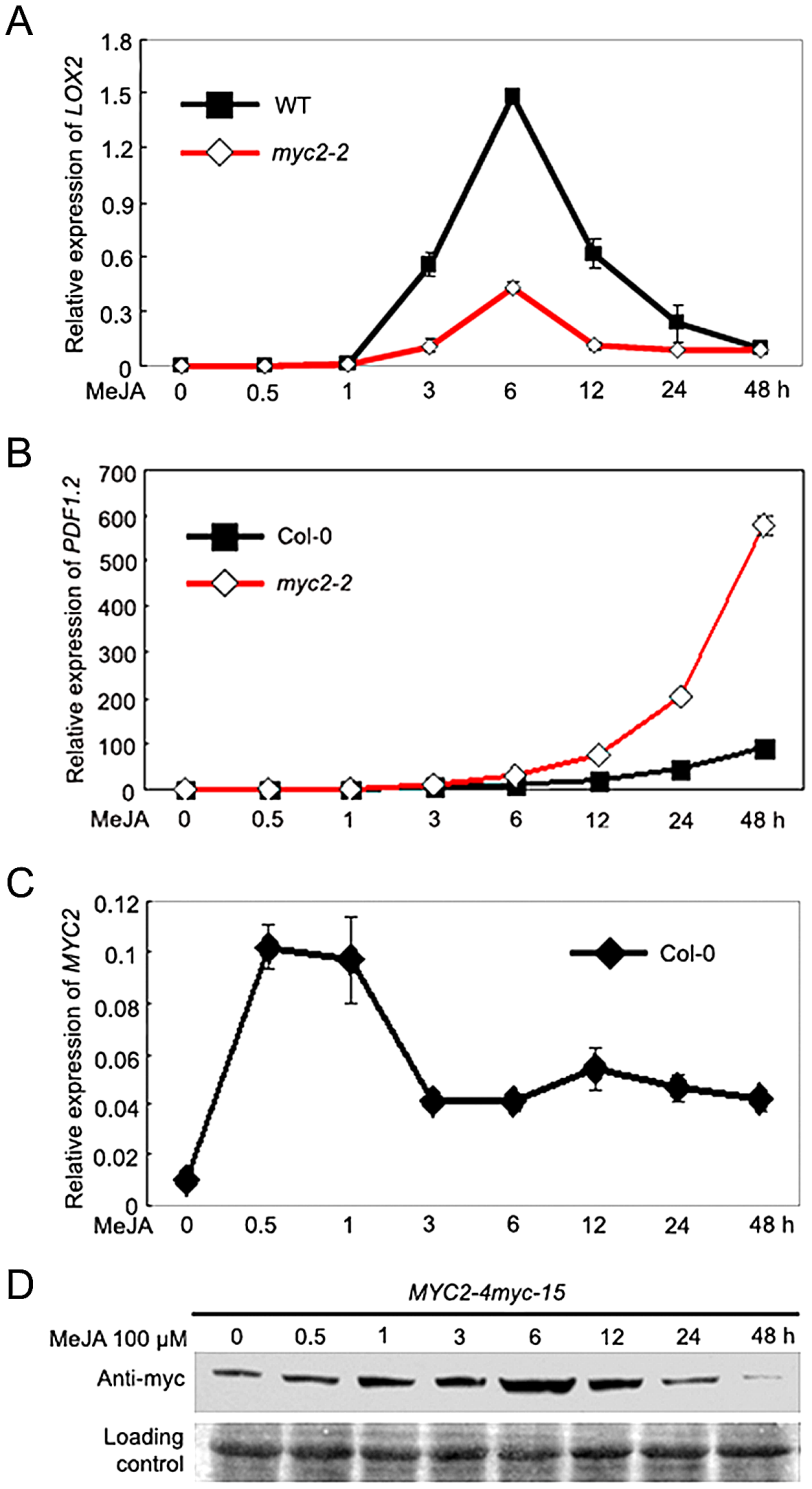
Temporal Correlation of JA-Induced Expression of *LOX2* and *PDF1.2* with JA-Induced Accumulation of the MYC2 Protein. (A–C) Time-course expression of *LOX2* (A), *PDF1.2* (B) and *MYC2* (C) in response to MeJA treatment. Seven-day-old seedlings were treated with 100 µM MeJA for indicated times before total RNAs were extracted for qRT-PCR assays. Values are mean ± SD of three technical replicates. (D) Time-course accumulation of the MYC2-myc fusion protein in MeJA-treated *MYC2-4myc-15* seedlings. Seedlings treatment was performed as in (A–C) and the MYC2-myc fusion protein was detected with an anti-myc antibody. Ponceau S staining of RbcS served as a loading control. Each experiment was repeated for at least three times with similar results.

To investigate the mechanism by which MYC2 differentially regulates distinct subsets of JA responses, we followed the time-course of MeJA-induced expression of wound- and pathogen-responsive genes in wild type (WT) and the *myc2-2* mutant [2]. For this analysis, we select *LIPOXYGENASE2* (*LOX2*) as a representative marker gene for wound response [21] and the plant defensin gene *PDF1.2* as a representative marker gene for pathogen response [22]. Quantitative real-time PCR (qRT-PCR) assays indicated that, in MeJA-treated WT seedlings, *LOX2* mRNA levels showed a pronounced increase at 3 h and reached to a maximum at 6 h; *LOX2* mRNA levels then showed a tendency of reduction and returned to basal levels at 48 h after MeJA treatment (Figure 1A). Parallel experiments indicated that, in MeJA-treated WT seedlings, *PDF1.2* mRNA remained at basal levels until 12 h and reached to a maximum at 48 h after treatment (Figure 1B). These results demonstrate that, JA-mediated induction of wound-responsive genes, which are positively regulated by MYC2, occurs relatively early. In contrast, JA-mediated induction of pathogen-responsive genes, which are negatively regulated by MYC2, occurs relatively late.

We then examined JA-induced accumulation of MYC2 at both mRNA and protein levels. As shown in Figure 1C, *MYC2* mRNA levels quickly reached to a maximum at 0.5 h after MeJA treatment then showed a tendency of reduction in the duration of the experiment. We then used the *MYC2-4myc-15* plants, which express a functional MYC2-4myc fusion protein (Figure S1), to examine the MeJA-induced accumulation kinetics of the MYC2 fusion protein. As shown in Figure 1D, upon MeJA treatment, the MYC2-myc fusion protein showed an obvious induction at 0.5 h, maintained at very high level from 3 h to 12 h, and exhibited a tendency of reduction from 24 h to 48 h (Figure 1D). Therefore, high accumulation of the MYC2 protein correlates with peaked expression of early wound-responsive genes, whereas low accumulation of the MYC2 protein correlates with peaked expression of late pathogen-responsive genes. These results implicate that temporal regulation of MYC2 protein accumulation is important for its function.

### The Negative Regulation of *PDF1.2* by MYC2 Is Mediated by Direct Suppression of *ORA59*

**Figure 2:**
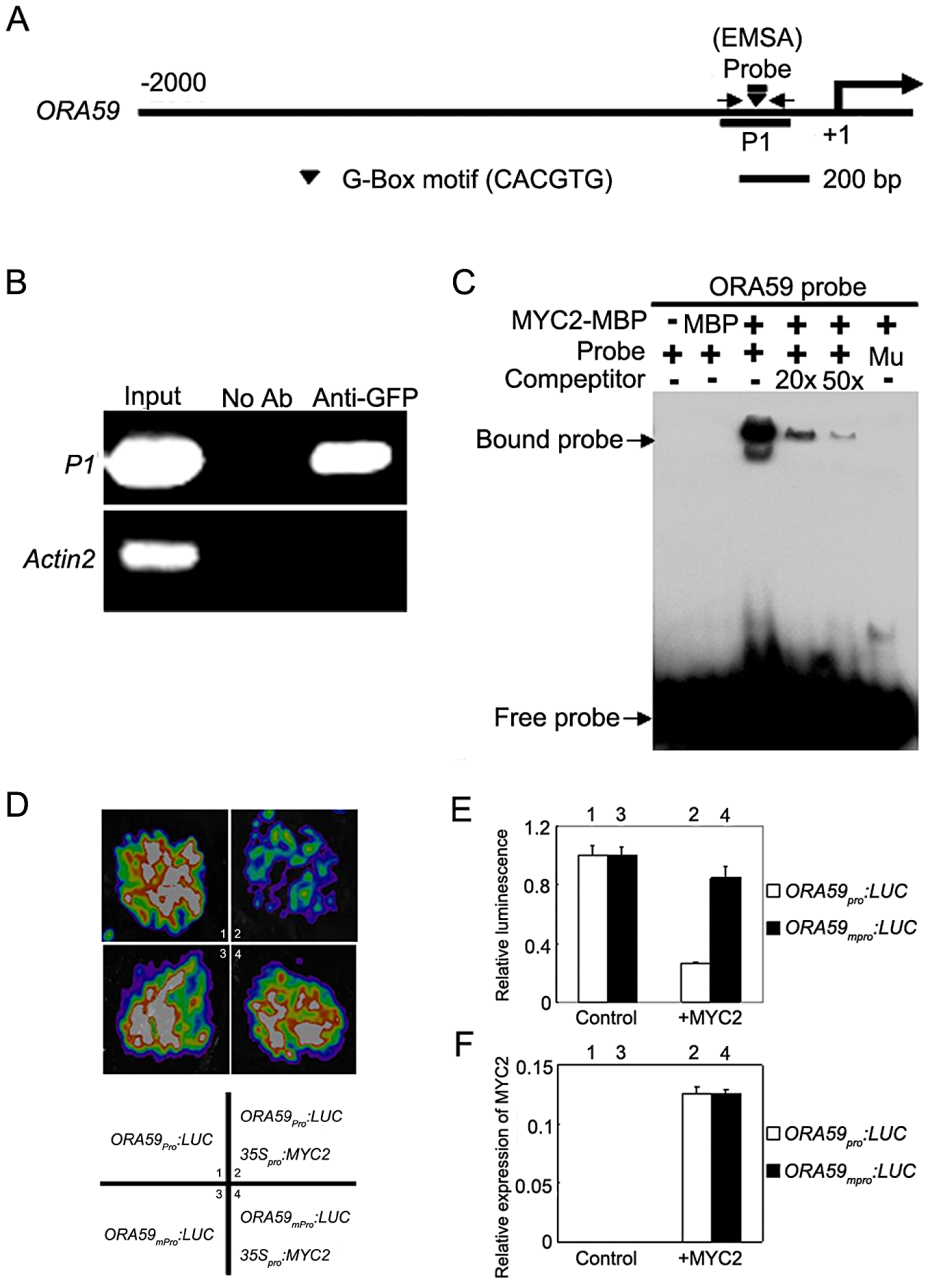
MYC2 Represses the Expression of *ORA59* by Directly Interacting with Its Promoter. (A) Schematic diagram of the *ORA59* promoter showing the potential MYC2 binding sites (black triangle), DNA fragment (P1) used for ChIP, and probe used for EMSA. The sequence 2000 bp upstream of the start site and part of the coding sequence of *ORA59* is shown. The transcriptional start site (ATG) is shown at position +1. (B) Enrichment of the indicated DNA fragment (P1) following ChIP using anti-GFP antibody. Chromatin of transgenic plant expressing *35S_pro_:MYC2-GFP* was immuno-precipitated with an anti-GFP antibody, and the presence of the indicated DNA in the immune complex was determined by RT-PCR. The *ACTIN2* promoter fragment was used as a negative control. The experiment was repeated three times with similar results. (C) EMSA showing that the MYC2-MBP fusion protein binds to the DNA probes of *ORA59* in vitro. Biotin-labeled probes were incubated with MYC2-MBP protein, and the free and bound DNAs (arrows) were separated in an acrylamide gel. As indicated, unlabeled probes were used as competitors. Mu, mutated probe in which the 5′-CACGTG-3′ motif was replaced by 5′-AAAAAA-3′. (D) Transient expression assays showing that MYC2 represses the expression of *ORA59*. Representative images of *N. benthamiana* leaves 72 h after infiltration are shown. The bottom panel indicates the infiltrated constructs. (E) Quantitative analysis of luminescence intensity in (D). Values are mean ± SD of five independent determinations. (F) qRT-PCR analysis of *MYC2* expression in the infiltrated leaf areas shown in (D). Total RNA was extracted from leaves of *N. benthamiana* coinfiltrated with the indicated constructs. Values are mean ± SD of five independent determinations.

Previous studies revealed two mechanisms by which MYC2 activates the expression of early wound-responsive genes including *LOX2*, *TAT1* and *VSP1*. First, MYC2 activates *LOX2* and *TAT1* transcription by directly binding to their promoters [23]; Second, MYC2 directly activates the expression of intermediate transcription factors such as ANAC019 and ANAC055 [24], which, in turn, activate the expression of *VSP1* [25]. It was reported that the effect of MYC2 on the transcription of pathogen-responsive genes is mainly achieved through direct regulation of a spectrum of intermediate transcription factors [3]. For example, it was shown that the negative regulation of *PDF1.2* expression by MYC2 is achieved through directly suppression of EHYLENE RESPONSE FACTOR 1 (ERF1) [3]. We provided several lines of evidence supporting that, in addition to *ERF1*, the transcription factor OCTADECANOID-RESPONSIVE ARABIDOPSIS AP2/ERF-domain protein 59 (ORA59), which directly binds the promoter of *PDF1.2* [26], is also involved in MYC2-mediated suppression of *PDF1.2* expression. First, MeJA-induced expression levels of *ORA59* was dramatically increased in *myc2-2* than those in WT (Figure S2), indicating that MYC2 negatively regulates MeJA-induced expression of *ORA59*. Second, chromatin immunoprecipitation (ChIP) assays using the previously described *35S_pro_:MYC2-GFP* plants [27] indicated that MYC2 associates with a G-box hexamer ‘CACGTG’ in the *ORA59* promoter (Figure 2A and 2B). Third, DNA electrophoretic mobility shift assays (EMSA) indicated that a MYC2-maltose binding protein (MBP) fusion protein binds the *ORA59* promoter sequence in a G-box-dependent manner (Figure 2A and 2C). Finally, using the transient expression assay of *Nicotiana benthamiana* leaves, we verified the repression effect of MYC2 on the expression of a reporter containing the *ORA59* promoter fused with the firefly luciferase gene (*LUC*) (Figure 2D–2F). Together, these results indicate that ORA59 is a member of the intermediate transcription factors involved in MYC2-mediated suppression of *PDF1.2* expression.

### MYC2 Is Subjected to Proteasome-Mediated Degradation

**Figure 3:**
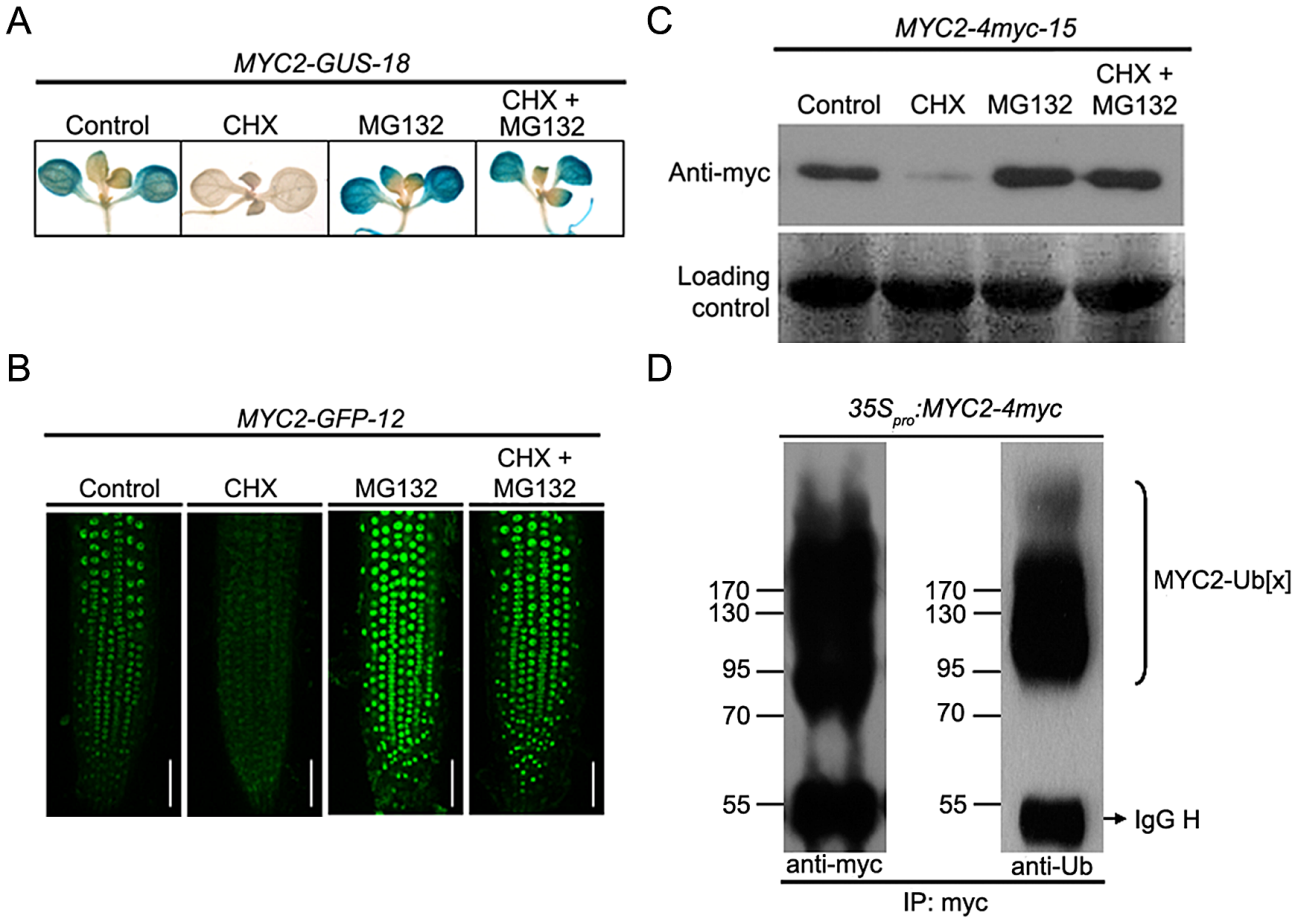
MYC2 Is Subjected to Proteasome-Mediated Degradation. (A) Seven-day-old seedlings of *MYC2-GUS-18* were treated with 100 µM CHX and/or 50 µM MG132 for 6 h before GUS staining was visualized. (B) Seven-day-old seedlings of *MYC2-GFP-12* were treated with 100 µM CHX and/or 50 µM MG132 for 6 h before the fluorescence were monitored. Bars = 50 µm. (C) Seven-day-old seedlings of *MYC2-4myc-15* were treated with 100 µM CHX and/or 50 µM MG132 for 6 h before total proteins were extracted for western blotting using an anti-myc antibody. Ponceau S staining of RbcS served as a loading control. (D) *N. benthamiana* leaves expressing the *35_pro_:MYC2-4myc* transgene were treated with 50 µM MG132 for 12 h. Protein extracts were immunoprecipitated using an anti-myc antibody and then were analyzed by western blotting using anti-myc or anti-ubiquitin (Ub) antibodies. Each experiment was repeated for at least three times with similar results.

The existence of a temporal correlation between MYC2 protein accumulation and its function to differentially regulate wound response and pathogen response suggests that protein stability may play a role in MYC2 regulation. To test that MYC2 may be subjected to proteolysis *in planta*, we generated *35S_pro_:MYC2-GUS*, *35S_pro_:MYC2-GFP* and *35S_pro_:MYC2-4myc* constructs and introduced them into the *myc2-2* mutants. The resulting stable transgenic lines including *MYC2-GUS-18*, *MYC2-GFP-12* and *MYC2-4myc-15*, which expressed comparable transcript levels of the respective transgene and rescued the JA-insensitive phenotype of the *myc2-2* mutant (Figure S1), were selected for protein stability and functional analysis. Upon application of cycloheximide (CHX), an inhibitor of *de novo* protein synthesis, GUS activity of *MYC2-GUS-18* seedlings (Figure 3A) or GFP fluorescence of *MYC2-GFP-12* seedlings (Figure 3B) were largely reduced. In line with a previous observation that CHX up-regulates *MYC2* transcripts [28], we showed that the mRNA levels of the *MYC2-GUS* or *MYC2-GFP* transgenes were actually increased in CHX-treated transgenic seedlings (Figure S3). These results eliminate the effect of transcriptional regulation on the protein abundance of the *MYC2-GUS* or *MYC2-GFP* fusions and support that the MYC2-reporter fusion proteins are unstable. Addition of the proteasome inhibitor MG132 to the transgenic seedlings, which barely affects the mRNA levels of the transgenes (Figure S3), lead to increased signal intensity of GUS staining (Figure 3A) or GFP fluorescence (Figure 3B). Furthermore, co-treatment with MG132 and CHX largely blocked the effect of CHX (Figure 3A and 3B). These results indicate that degradation of the MYC2-GUS or MYC2-GFP fusions requires the proteasome activity. Similarly, protein gel blot assays using *MYC2-4myc-15* seedlings indicated that, whereas addition of CHX led to reduced MYC2-4myc levels, addition of MG132 led to increased MYC2-4myc levels and, the CHX effect was sufficiently suppressed by MG132 (Figure 3C).

As ubiquitination is a prerequisite for protein degradation by the 26S proteasome, we asked whether we could detect the ubiquitinated form of MYC2. For this experiment, the *35S_pro_:MYC2-4myc* construct was transferred into *N. benthamiana* leaves with the well-established agroinfiltration system [29]. Protein extracts from the agroinfiltrated leaves were immunoprecipitated with an anti-myc antibody and examined via western blotting using an anti-myc antibody. As shown in Figure 3D, in addition to the band of expected size for the MYC2-4myc protein, a smear of bands corresponding to larger molecules, which show the feature of ubiquitinationed forms of the MYC2-4myc fusion protein, were also detected. Indeed, when the same samples were immuno-analyzed with an anti-ubiquitin antibody, the high molecular size bands could be recognized by the anti-ubiquitin antibody, confirming that these additional bands were ubiquitinated forms of the MYC2-4myc protein (Figure 3D). Together, these findings led us to a conclusion that MYC2 is subjected to UPS-dependent proteolysis.

### Degradation of MYC2 Is Important for Its Function to Regulate Gene Transcription

**Figure 4:**
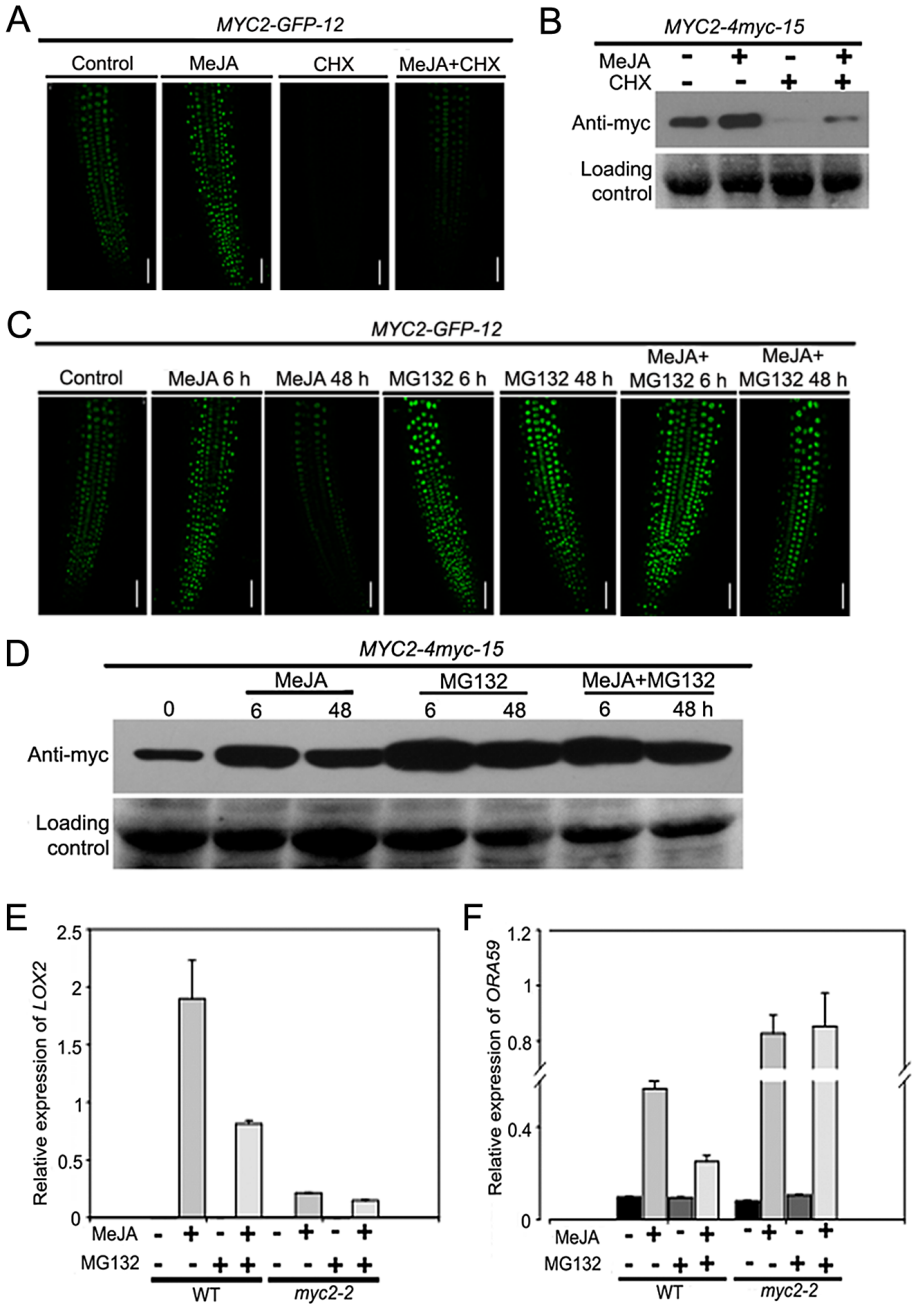
JA-Induced Transcription of MYC2 Target Genes Requires the Proteasome. (A) Seven-day-old seedlings of *MYC2-GFP-12* were treated with 100 µM MeJA and/or 100 µM CHX for 6 h, root tissues were then examined by fluorescence microscopy. Bars = 50 µm. (B) Seven-day-old seedlings of *MYC2-4myc-15* were treated with 100 µM MeJA and/or 100 µM CHX for 6 h. Total protein extracts were analyzed by western blotting using anti-myc antibody. Ponceau S staining of RbcS served as a loading control. (C) Seven-day-old seedlings of *MYC2-GFP-12* were treated with 100 µM MeJA and/or 50 µM MG132 for indicated times, root tissues were then examined by fluorescence microscopy. Bars = 50 µm. (D) Seven-day-old seedlings of *MYC2-4myc-15* plants were treated with 100 µM MeJA and/or 50 µM MG132 for indicated times. Total protein extracts were analyzed by western blotting using an anti-myc antibody. Ponceau S staining of RbcS served as a loading control. (E) and (F) qRT-PCR analysis of MeJA-induced expression of *LOX2* (E) and *ORA59* (F) in WT and *myc2-2*. Seven-day-old seedlings were treated with 100 µM MeJA and/or 50 µM MG132 for 6 h (E) or 48 h (F) and RNAs were extracted for qRT-PCR assays. Values are mean ± SD of three technical replicates. Each experiment was repeated at least three times with similar results.

To test that MYC2 degradation is a part of the JA signaling, the above-described *MYC2-GFP-12* seedlings were treated with MeJA in the absence or presence of CHX. In the absence of CHX, GFP fluorescence showed a marked increase at 6 h following MeJA treatment and, MeJA-induced elevation of GFP fluorescence was dramatically decreased by the addition of CHX (Figure 4A). Similarly, as revealed by western blot assays, treatment of the *MYC2-4myc-15* seedlings with MeJA alone led to a strong elevation of the MYC2-4myc fusion protein and MeJA-induced increase of the fusion protein was markedly reduced by CHX (Figure 4B). Considering that addition of CHX actually showed an increasing effect on MeJA-mediated increase of the mRNA levels of the *MYC2-GFP* or *MYC2-myc* transgenes (Figure S4), these results support that UPS-mediated degradation of MYC2 occurs during JA signaling.

To substantiate this observation, we monitored the MeJA-mediated change of the MYC2-GFP fluorescence of the *MYC2-GFP-12* seedlings in the absence or presence of the UPS inhibitor MG132. Upon treatment with MeJA itself, GFP signal peaked at 6 h and returned to basal levels at 48 h (Figure 4C). In the presence of MG132, however, MeJA-induced increased of GFP signal was strengthened at both 6 h and 48 h, indicating that MeJA-induced fluctuations of the GFP signal were abolished by MG132 (Figure 4C). This MG132 effect on MeJA-induced fluctuations of the MYC2-GFP fluorescence provides us a facile assay to investigate the mechanism underlying MYC2 degradation. Similarly, as revealed by western blot assays, MeJA-induced fluctuations of the MYC2-4myc fusion protein levels were also abolished by MG132 (Figure 4D).

To evaluate the effect of MYC2 degradation on its transcription activity, we examined whether MG132 affects MeJA-induced expression of *LOX2* and *ORA59*, marker genes of JA-induced wound and pathogen responses that were direct targets of MYC2. In line with the notion that MYC2 positively regulates wound response, MeJA-induced expression levels of *LOX2* were largely reduced by the *myc2-2* mutation (Figure 4E). Whereas treatment with MG132 alone resulted in undetectable MYC2-dependent induction of *LOX2* expression, the MeJA-mediated induction of this gene was strongly inhibited in the presence of MG132 (Figure 4E), indicating that MeJA-induced activation of *LOX2* expression requires both MYC2 and the proteasome activity. Similarly, MG132 itself showed negligible effect on MYC2-dependent regulation of *ORA59* expression and this proteasome inhibitor strongly weakened the MeJA-induced activation of *ORA59* expression in WT plants (Figure 4F). Consistent with a negative effect of MYC2 on JA-mediated induction of *ORA59* expression, MeJA-induced expression levels of *ORA59* were already high in the *myc2-2* mutant, and addition of MG132 showed minor, if any, effect on MeJA-induced expression of *ORA59* in this mutant (Figure 4F). Collectively, these results demonstrate that proteasome activity is required for the MYC2 function to differentially regulate wound response and pathogen response.

### A 12-Amino-Acid Destruction Element of the MYC2 Protein Overlaps with Its TAD

**Figure 5:**
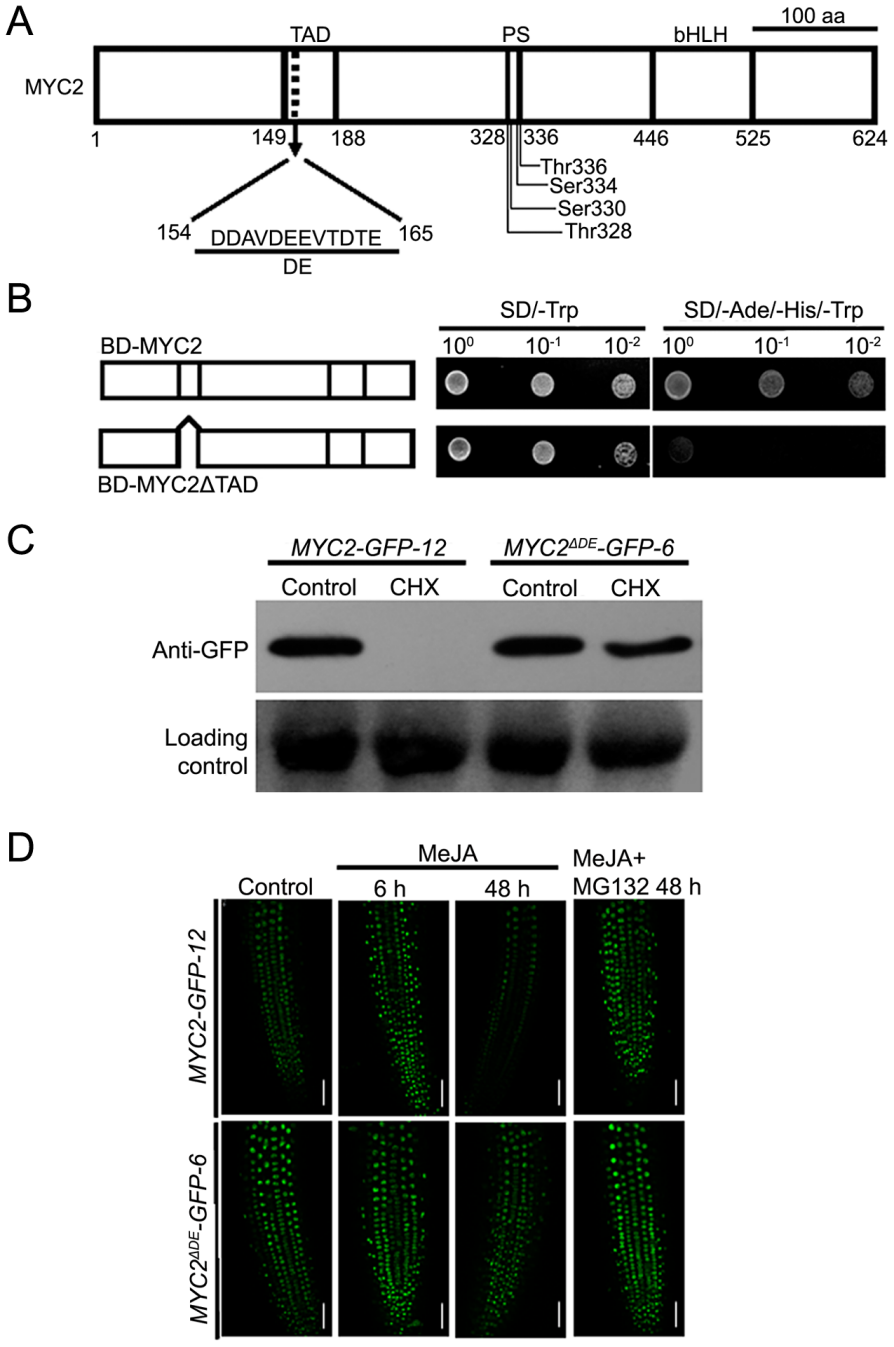
The Destruction Element of the MYC2 Protein Overlaps with Its TAD. (A) Schematic representation of MYC2 structural domains. TAD: transcriptional activation domain. DE: destruction element. PS: predicted phosphorylation sites. bHLH: basic helix-loop-helix domain. (B) Yeast assays showing that the activation domain of MYC2 locates in amino acid from 149 to 188. Based on the schematic protein structure of MYC2 (Figure 4A), full length MYC2 or MYC2ΔTAD were tested for transcriptional activation. The right panels shows one-tenth and one-hundred dilution yeast growth in media. (C) Seven-day-old seedlings of *MYC2-GFP-12* and *MYC2^ΔDE^-GFP-6* were treated with 100 µM CHX for 6 h. Total protein was analyzed by western blotting using an anti-GFP antibody. Ponceau S staining of RbcS served as a loading control. (D) Seven-day-old seedlings of *MYC2-GFP-12* and *MYC2^ΔDE^-GFP-6* were treated with 100 µM MeJA and/or 50 µM MG132 for indicated times, root tissues were examined by fluorescence microscopy. Bars = 50 µm. Each experiment was repeated at least three times with similar results.

Our findings that the proteasome activity is important for the MYC2 function suggest that MYC2 proteolysis is tightly linked with its transcriptional activity. It is generally considered that the N-terminal part of MYC2 is important for its transcriptional activity [3], [4], [12], but the transcriptional activation domain (TAD) of MYC2 has not been identified based on experimental studies. We used the MATCHMAKER GAL4-based Two-Hybrid System 3 (Clontech) to define the TAD of MYC2. The expressed proteins in yeast strains were analyzed by immunoblot experiments (Figure S5). In these assays, we found that MYC2 has strong transcriptional activation activity whereas MYC2ΔTAD, in which amino acids from 149 to 188 of MYC2 were deleted, dose not (Figure 5A–5B), indicating that the domain from amino acid 149 to 188 could be the TAD of MYC2.

In the mammalian system, it is a general phenomenon that the destruction elements (DE), which are usually acidic, overlap closely with the TADs of unstable transcription factors [18], [28], [30]. Indeed, our sequence analysis of the MYC2 TAD region identified a 12-amino-acid element (MYC2^154–165^) that is enriched in acidic amino acids (Figure 5A). To test that this acidic domain may function as a degron of MYC2, we generated a DE deletion construct of MYC2 and introduced it into the *myc2-2* mutant. Among the resulted transgenic plants, the line *MYC2^ΔDE^-GFP-6* was selected for further analysis. As shown in Figure S6, *MYC2^ΔDE^-GFP-6* plants and the above-described *MYC2-GFP-12* plants showed comparable transcript levels of the respective transgenes. Whereas blocking protein synthesis with CHX strongly reduced the *MYC2-GFP* fusion protein in *MYC2-GFP-12* plants, CHX showed minor reduction effect on the *MYC2^ΔDE^-GFP* fusion protein in *MYC2^ΔDE^-GFP-6* plants (Figure 5C), indicating that deletion of the DE indeed affects the protein stability of MYC2. Next, through monitoring the MG132 effect on JA-induced fluctuations of GFP fluorescence as an assay, we found that, in the absence or presence of MG132, the MeJA-induced change of GFP fluorescence was largely abolished in *MYC2^ΔDE^-GFP-6* plants (Figure 5D). In summary, deletion of the 12-amino-acid element of MYC2 produces a protein remains stable support a scenario that the DE element we identified functions as a degron of MYC2.

### The Destruction Element Is Required for the MYC2 Function to Regulate Gene Transcription

**Figure 6:**
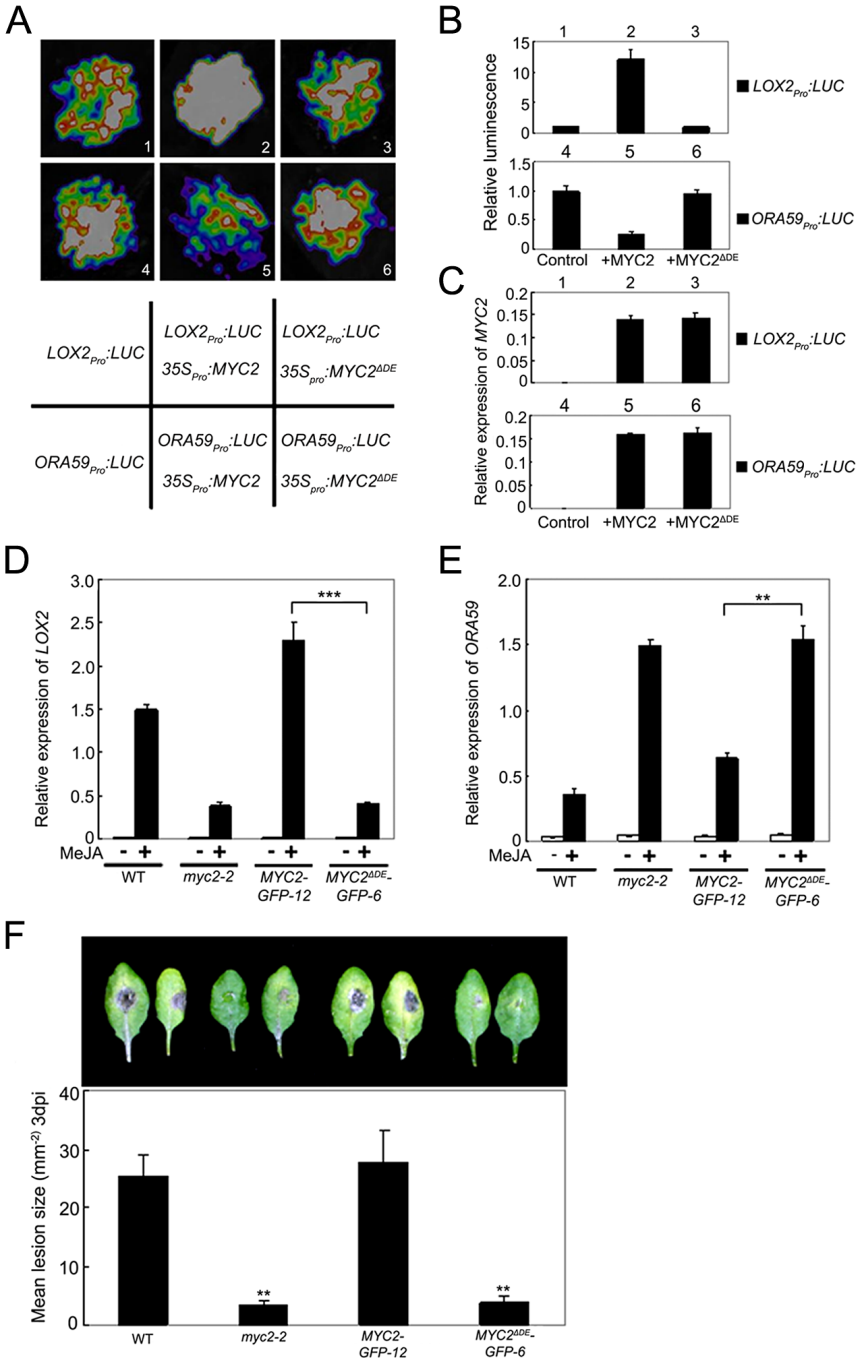
The Destruction Element of MYC2 Is Important for Its Transcription Activity. (A) Transient expression assays showing that *MYC2^ΔDE^* failed to regulate the expression of *LOX2* and *ORA59*. Luminescence imaging of *N. benthamiana* leaves is shown 72 h after coinfiltration with the constructs indicated in the bottom panel. (B) Quantitative analysis of luminescence intensity in (A). Values are mean ± SD of five independent determinations. (C) qRT-PCR analysis of *MYC2* expression in (A). Total RNA was extracted from leaves of *N. benthamiana* coinfiltrated with the constructs in (A). Values are mean ± SD of three technical replicates. (D) and (E) qRT-PCR analysis of MeJA-induced expression of *LOX2* (D) and *ORA59* (E). Seven-day-old seedlings of the indicated genotypes were treated with 100 µM MeJA for 6 h (D) or 48 h (E) before tissues were harvested for RNA extraction. Values are mean ± SD of three technical replicates. Asterisks represent Student's t-test significance between pairs indicated with brackets (**, P<0.01). (F) Detached leaves from 4-week-old plants of the indicated genotypes were inoculated with *B. cinerea* spores for 3 d. Symptoms on rosette leaves were shown and lesion size (mm^−2^) was measured. Values are mean ± SD of 20 leaves from 20 plants. Asterisks denote Student's t-test significance compared with WT plants: **, P<0.01. Each experiment was repeated at least three times with similar results.

ChIP-PCR assays using *MYC2^ΔDE^-GFP-6* plants indicated that deletion of the DE does not affect the binding capacity of MYC2 to the promoter of *ORA59* (Figure S7). To test that the DE may affect MYC2-directed activation of wound responsive genes, we used a transient assay to compare the activation effect of MYC2 or MYC2^ΔDE^ on the expression of *LOX2_pro_:LUC*, a reporter containing the *LOX2* promoter fused with the *LUC* gene. Co-expression of *LOX2_pro_:LUC* with *35S_pro_:MYC2* led to an obvious increase of luminescence intensity (Figure 6A–6C), indicating that *35S_pro_:MYC2* activates the expression of *LOX2_pro_:LUC*. In contrast, the *35S_pro_:MYC2^ΔDE^* construct failed to activate the expression of *LOX2_pro_:LUC* (Figure 6A–6C). These results support that the DE is required for the MYC2 function to activate the expression of *LOX2*.

To confirm this *in planta*, we examined JA-induced expression of *LOX2* in *MYC2-GFP-12*, *MYC2^ΔDE^-GFP-6*, *myc2-2* and WT plants. MeJA-induced expression levels of *LOX2* in *MYC2-GFP-12* plants were much higher than those in the WT (Figure 6D), indicating that the *35S_pro_:MYC2-GFP* construct rescued the JA-insensitive phenotype of *myc2-2* in term of JA-induced *LOX2* expression. In *MYC2^ΔDE^-GFP-6* plants, however, MeJA-induced expression levels of *LOX2* were essentially comparable to those in the *myc2-2* mutant (Figure 6D), indicating that the *35S_pro_:MYC2^ΔDE^-GFP* construct failed to rescue the *myc2-2* mutant phenotype. These results support that the DE is important for the MYC2 function to activate the transcription of wound-responsive genes.

Similarly, in a transient assay of *N. benthamiana* leaves, we showed that DE is also required for the MYC2 function to repress the expression of *ORA59_pro_:LUC*, a reporter containing the *ORA59* promoter fused with *LUC* (Figure 6A–6C). Comparison of JA-induced expression levels of *ORA59* in *MYC2-GFP-12*, *MYC2^ΔDE^-GFP-6*, *myc2-2* and WT plants indicated that, the *35S_pro_:MYC2-GFP* construct, but not the *35S_pro_:MYC2^ΔDE^-GFP* construct, rescued the *myc2-2* mutant phenotype in MeJA-induced *ORA59* expression (Figure 6E). Given that MYC2 negatively regulates pathogen response, a featured phenotype of *myc2-2* is that this mutant is more resistant than its WT counterpart to the necrotrophic pathogen *Botrytis cinerea* [4]. Our pathogen response assays revealed that, whereas *MYC2-GFP-12* plants showed a similar performance as WT plants, *MYC2^ΔDE^-GFP-6* plants showed a similar performance as *myc2-2* plants (Figure 6F). Collectively, these results lead us to a conclusion that the DE and TAD of MYC2 are functionally connected.

### MYC2 Phosphorylation at Thr328 Facilitates Its Turnover

**Figure 7:**
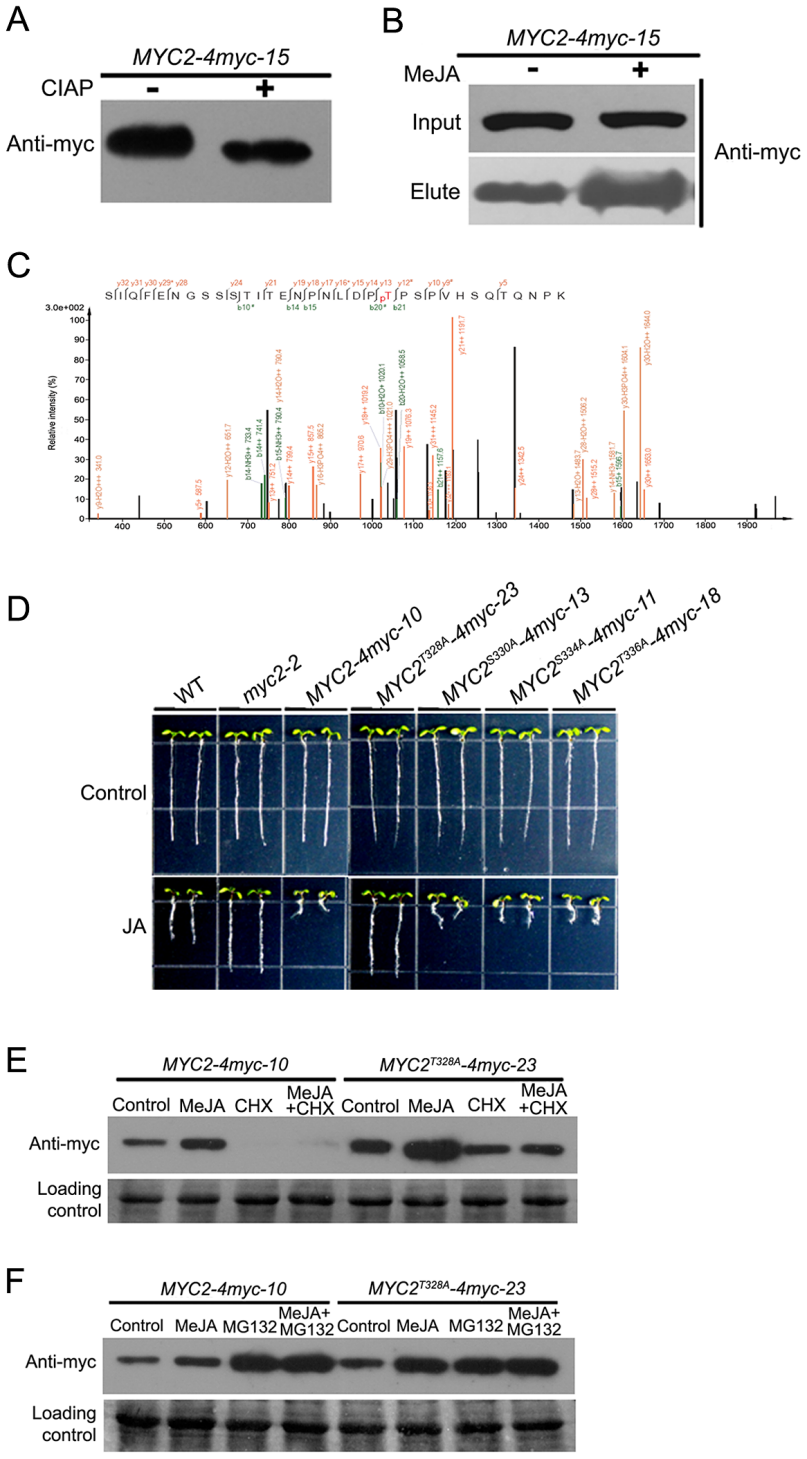
Phosphorylation of MYC2 at Thr328 Affects Its Turnover. (A) Total protein of *MYC2-4myc-15* plants was extracted and immunoprecipitated with an anti-myc antibody. Immunoprecipitated proteins were treated with alkaline phosphatase for 30 min then analyzed by western blotting using an anti-myc antibody. (B) *MYC2-4myc-15* transgenic plants were treated with or without 100 µM MeJA for 6 h. Total proteins containing equal amount of MYC2-4myc were loaded on a column that specifically binds phospho-proteins. Bound proteins were eluted and the amount of MYC2-4myc determined by western blotting with an anti-myc antibody. (C) Identification of MYC2 phosphorylation at Thr328 by mass spectrometry. Shown is a collision-induced dissociation mass spectrum of the phosphopeptide SIQFENGSSSTITENPNLDP(pT)PSPVHSQTQNPK (+3 charged, *m/z* 1212.22). The C-terminal fragments (*y* ions) are colored orange and the N-terminal fragments (*b* ions) are colored green. ^*^ and ^#^ indicate fragment ions with a neutral loss of phosphoric acid or H_2_O, respectively. (D) Root growth inhibition assay of the transgenic plants as indicated. Seeds were germinated on 1/2 MS medium with or without 20 µM MeJA after 3 d stratification; photos were taken 8 d after germination. (E) *MYC2-4myc-10* and *MYC2^T328A^-4myc-23* plants were treated for 6 h with 100 µM MeJA and/or 100 µM CHX. Total protein was analyzed by western blotting using an anti-myc antibody. Ponceau S staining of RbcS served as a loading control. (F) *MYC2-4myc-10* and *MYC2^T328A^-4myc-23* plants were treated for 6 h with 100 µM MeJA and/or 50 µM MG132. Total protein was analyzed by western blotting using anti-myc antibody. Ponceau S staining of RbcS served as a loading control.

Targeting substrates to the proteasome is often regulated by post-translational modifications, such as phosphorylation. To test if MYC2 is phosphorylated, protein extracts from the *MYC2-4myc-15* transgenic plants were immunoprecipitated and treated with calf intestinal alkaline phosphatase (CIAP). We found that CIAP treatment led to a slightly faster migration of the MYC2-4myc fusion protein, implying that the slower migrating form of the fusion protein was phosphorylated (Figure 7A). Next, we treated *MYC2-4myc-15* plants without or with MeJA, extracted total protein and applied the extracts onto a column that specifically binds phosphorylated proteins. A protein gel blot assay was performed to make sure that the amount of the MYC2-4myc protein in control and MeJA-treated samples was comparable (Figure 7B). Western blot assays indicated that the MYC2-4myc fusion protein could bind to the column and, importantly, MeJA treatment led to a substantial increase of proteins bound to the column (Figure 7B). These results implicate that MYC2 is phosphorylated *in vivo* and that MYC2 phophorylation is under the regulation of the JA signal.

An examination of MYC2 protein sequence revealed a cluster of four potential phosphorylation residues including Thr328, Ser330, Ser334 and Thr336 (Figure 5A). Mass spectrometric analysis of MYC2 immunoprecipitated from the *MYC2-4myc-15* seedlings revealed phosphorylation at Thr328 (Figure 7C). To find out the physiological function of the confirmed and potential phosphorylation sites, phosphorylation defective forms of the MYC2-4myc fusion protein carrying serine/threonine to alanine mutations (i.e., MYC2^T328A^, MYC2^S330A^, MYC2^S334A^, MYC2^T336A^) were introduced into the *myc2-2* mutant under the 35S promoter. Transgenic lines *MYC2^T328A^-4myc-23*, *MYC2^S330A^-4myc-13*, *MYC2^S334A^-4myc-11* and *MYC2^T336A^-4myc-18*, in which the expression levels of the respective transgenes were comparable to those of the *MYC2-4myc-10* plants (Figure S8), were selected for further analysis. JA-induced root growth inhibition assays indicated that *MYC2^T328A^-4myc-23*, but not the rest of the transgenic lines, showed a JA-insensitive phenotype like *myc2-2* (Figure 7D), revealing that the T328A mutation, but not the other three mutations, affects the MYC2 protein function. These results support that Thr328 is an *in vivo* phosphorylation site of MYC2.

To determine whether Thr328 phophorylation affects MYC2 turnover, *MYC2-4myc-10* and *MYC2^T328A^-4myc-23* seedlings were treated with MeJA and the abundance of the fusion proteins were examined. We found that the basal and MeJA-induced accumulation levels of MYC2^T328A^-4myc were higher than those of the MYC2-4myc and, importantly, that the CHX-mediated reduction of the fusion protein accumulation was abolished in *MYC2^T328A^-4myc-23* seedlings (Figure 7E). Similarly, in the absence or presence of MeJA, MG132-mediated increase of the fusion protein accumulation was also abolished in *MYC2^T328A^-4myc-23* seedlings (Figure 7F). Together, our findings that the MYC2^T328A^ mutation renders the MYC2-4myc fusion more stable support that phosphorylation of Thr328 facilitates the proteolysis of the MYC2 protein.

### Phosphorylation of MYC2 at Thr328 Is Coupled with Its Transcription Activity

**Figure 8:**
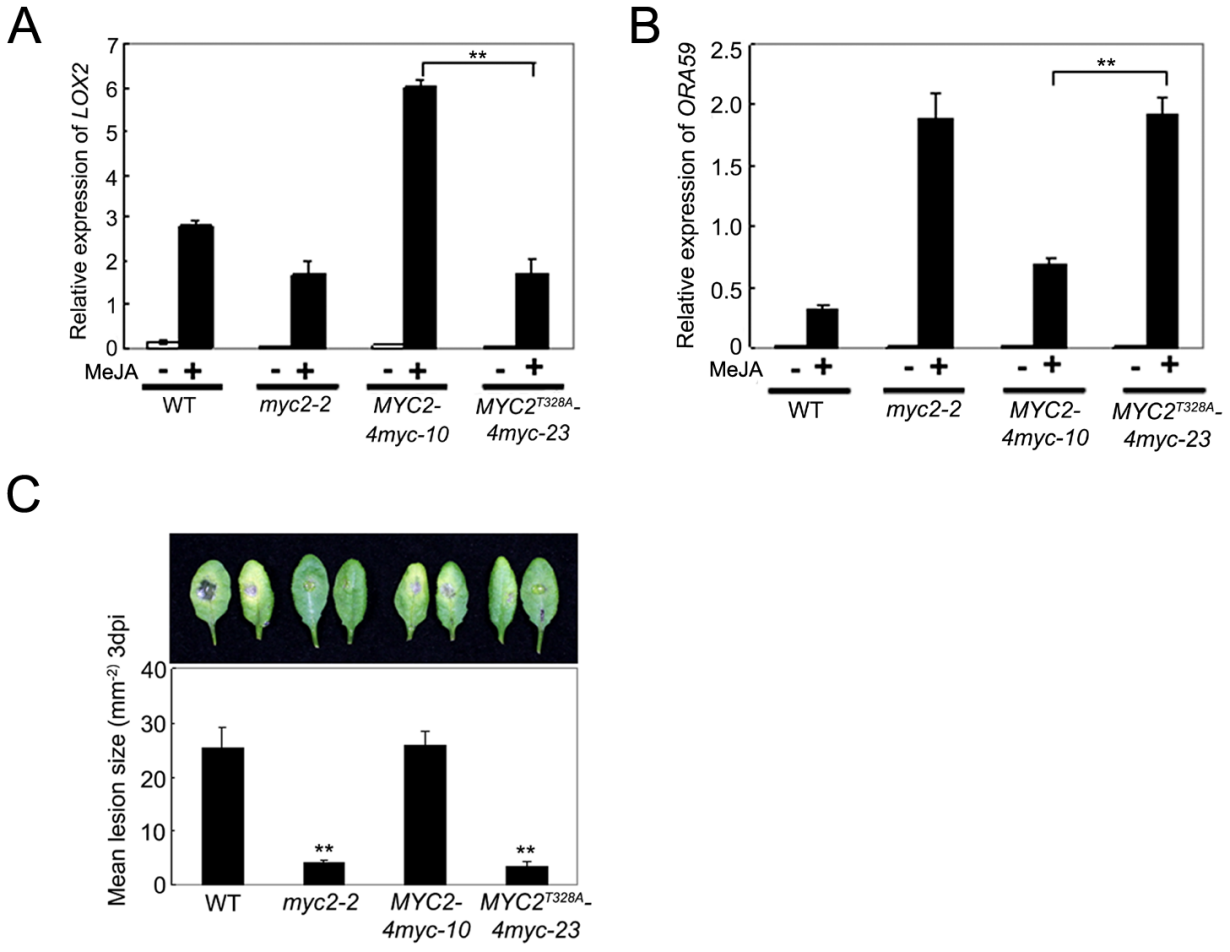
Phosphorylation of MYC2 at Thr328 Affect Its Transcription Activity. (A) and (B) qRT-PCR analysis of MeJA-induced expression of *LOX2* (A) and *ORA59* (B). Seven-day-old seedlings of the indicated genotypes were treated with 100 µM MeJA for 6 h (A) or 48 h (B) before tissues were harvested for RNA extraction. Values are mean ± SD of three technical replicates. Asterisks represent Student's t-test significance between pairs indicated with brackets (**, P<0.01). (C) Detached leaves from 4-week-old plants of the indicated genotypes were inoculated with *B. cinerea* spores for 3 d. Symptoms on rosette leaves were shown and lesion sizes (mm^−2^) were measured. Values are mean ± SD of 20 leaves from 20 plants. Asterisks denote Student's t-test significance compared with WT plants: **, P<0.01. Each experiment was repeated at least three times with similar results.

To test whether the MYC2^T328A^ mutation affects MYC2-dependent gene transcription, we compared MeJA-induced expression of *LOX2* and *ORA59* in *MYC2-4myc-10*, *MYC2^T328A^-4myc-23*, *myc2-2* and WT seedlings. As shown in Figure 8A, whereas MeJA-induced expression levels of *LOX2* in *MYC2-4myc-10* seedlings were strongly increased than those in WT seedlings, MeJA-induced expression levels of *LOX2* in *MYC2^T328A^-4myc-23* seedlings remained comparable to those in *myc2-2* seedlings. In parallel experiments, the MeJA-induced expression levels of *ORA59* in *MYC2-4myc-10* seedlings were substantially reduced compared to those in *myc2-2* seedlings, but the MeJA-induced expression levels of *ORA59* in *MYC2^T328A^-4myc-23* seedlings were essentially comparable to those in *myc2-2* seedlings (Figure 8B). In *B. cinerea* infection assays, the performance of *MYC2-4myc-10* seedlings is similar to that of the WT seedlings, whereas the performance of *MYC2^T328A^-4myc-23* seedlings is similar to that of the *myc2-2* seedlings (Figure 8C). Collectively, these results support that MYC2 phosphorylation at Thr328 is functionally coupled with its action to regulate JA-responsive gene transcription.

## Discussion

### Temporal Regulation of the MYC2 Protein Accumulation Facilitates Its Function to Differentially Regulate Different Aspects of JA–Mediated Immunity

Several lines of evidence hint the existence of protein regulation for the function of MYC2 in regulating JA-dependent plant immunity. For example, it has been shown that *MYC2* is upregulated by JA at the transcription level but, transgenic *Arabidopsis* plants overexpressing *MYC2* or its functional homolog in tomato (*Solanum lycopsicon*) did not show constant expression of defense genes without the JA signal [2], implying that a JA-dependent posttranscriptional modification of MYC2 is required for its function. Indeed, it was recently shown that the circadian-clock component TIME FOR COFFEE (TIC) interacts with and negatively regulates the protein accumulation of MYC2 [31]. A prominent action mode of MYC2 is that this transcription factor differentially regulates different subsets of JA-mediated immune responses. For example, MYC2 positively regulates the expression of early wound-responsive genes, whereas negatively regulates the expression of late pathogen-responsive genes [2]–[4], indicating that MYC2 can act both as a transcriptional activator and repressor. However, whether protein regulation of MYC2 itself is involved in its action to temporally activate or repress specific genes remains largely unknown. Therefore, one of the major challenges in understanding the action mechanisms of MYC2 is to uncover the regulation of this transcription factor at the protein level.

In this study, we found a temporal correlation between the MYC2 protein accumulation and its differential effects on the expression of wound responsive genes and pathogen responsive genes: high accumulation of the MYC2 protein correlates with peaked expression of the branch of early wound-responsive genes that are positively regulated by MYC2, whereas low accumulation of the MYC2 protein correlates with peaked expression of the branch of late pathogen-responsive genes that are negatively regulated by MYC2 (Figure 1). Clearly, the accumulation kinetics of the MYC2 protein during JA signaling facilitates its temporal activation or repression of specific subset of genes. In the context that inducible defense is an energy costly process and that plants have evolved the ability to precisely allocate limited resources in an attacker dependent manner [32], our findings support that MYC2 protein regulation plays an important role in resource management during JA-mediated plant immunity.

### Proteolysis of MYC2 Is Coupled with Its Transcription Activity

The existence of a temporal correlation between MYC2 protein accumulation and its function to differentially regulate wound response and pathogen response suggests that protein stability may play a role in MYC2 regulation. Indeed, we describe here that MYC2 is subjected to UPS-dependent proteolysis and demonstrate that UPS-dependent proteolysis of MYC2 is part of the JA signaling. It is well known that UPS-dependent proteolysis plays an important role in nearly every aspect of plant biology, including plant hormone signaling. In these instances UPS either degrades transcription factors to suppress transcription or degrades transcription repressors to activate gene expression [33]–[36]. Surprisingly, we provide here evidence that UPS-dependent proteolysis plays a counterintuitive role in the regulation of MYC2 during JA-mediated immune responses: First, UPS activity is essential for the MYC2 function to temporally activate or repress specific genes; Second, a 12-amino acid element in the TAD of MYC2 plays dual roles to signal proteolysis and to regulate transcription activity of MYC2. These data support that UPS-dependent turnover of MYC2 is coupled with its transcription activity during JA-mediated plant immunity. We further show that phosphorylation at Thr328 is important for both the proteolysis and the transcription activity of MYC2, indicating that UPS-mediated destruction of MYC2 is inherently linked to the way in which it stimulates gene transcription. In the plant kingdom, a similar regulatory mechanism was observed recently for the regulation of NONEXPRESSOR OF PATHOGENESIS-RELATED GENES (NPR1), a transcription coactivator involved in salicylic acid (SA)-mediated plant immunity [37].

Our finding that UPS-mediated proteolysis is involved directly and mechanistically in the regulation of MYC2 fits well with a scenario in the animal and yeast called “activation by destruction”, in which UPS-mediated proteolysis can activate the activity of the transcriptional regulators it destroys [13], [18]–[20], [30], [37]–[41]. This “activation by destruction” phenomenon has only been appreciated in recent years, but it appears to apply to an ever growing number of transcriptional regulators in animal and yeast [13], [19], [20]. Current hypotheses to explain the connection between transcription regulator destruction and function hold three main points: First, destruction of transcription factors is likely to be a direct consequence of their ability to activate transcription; Second, turnover of transcription factors occurs on the chromatin and during the process of gene induction; Third, turnover of transcription factors is often signaled by kinases that are integral parts of the transcriptional machinery [13], [38]. Based on these hypotheses and our findings in this study, we propose a model to explain how the UPS-dependent proteolysis stimulates the transcription activity of MYC2. On the chromatin and during JA-mediated induction process of MYC2 target genes, kinases associated with the transcriptional machinery serve to mark MYC2 as “spent”, trapping it in an inactive state. At the same time, these phosphorylation events bring the UPS machinery in and therefore destroy the “spent” MYC2 in situ, clearing the deck for promoter association with a “fresh” MYC2 molecule. In this model, the initial ‘pioneer round’ of transcription does not involve UPS-dependent proteolysis of MYC2. Proteolysis plays a positive role in transcription by allowing “fresh” MYC2 to access the promoter and therefore stimulates additional rounds of transcription. It will be interesting in future studies to prove that phosphorylation-coupled proteolysis of MYC2 occurs on the chromatin and to identify the kinases and E3 ligases involved in the phophorylation and proteolysis of MYC2. Regardless of these open questions, this study clearly demonstrates that phosphorylation-coupled proteolysis of transcription factors may be a common mechanism by which higher plants regulate gene transcription.

## Materials and Methods

### Plant Materials and Growth Conditions

*Arabidopsis thaliana* ecotype Columbia (Col-0) was used as wild type (WT). T-DNA insertion mutant *myc2-2* was previously described [1], [27].

*Arabidopsis* plants were grown in Murashige and Skoog (MS) media at 22°C with a 16-h-light/8-h-dark photoperiod (light intensity 120 µM photons m^−2^s^−1^) as previously described [42]. For MeJA-induced gene expression and protein accumulation assays, seedlings of indicated ages grown under 16-h light/8-h dark were treated with 100 µM MeJA. MeJA-treated seedlings were then transferred to continuous light for indicated times.

### DNA Constructs and Plant Transformation

The *35S_pro_:MYC2-GUS* was prepared by inserting PCR-amplified coding sequence of MYC2 and Glucuronidase (GUS) into the KpnI-SpeI sites and SalI-PstI sites of the binary vector pCAMBIA2300 under the control of cauliflower mosaic virus 35S promoter. The *35S_pro_:MYC2-GFP* and *35S_pro_:MYC2-4myc* constructs used in this study were previously described [1], [27]. To generate *35S_pro_:MYC2^ΔDE^-GFP* construct, coding sequence of MYC2 truncated DE domain was amplified with Gateway-compatible primers. The PCR product was cloned by pENTR Directional TOPO cloning kit (Invitrogen) and then recombined with the binary vector pGWB5 (35S promoter, C-GFP) [43]. The codon for Thr328 of MYC2 in *pENTR-MYC2* [1] was replaced with the amino acid encoding alanine using the TaKaRa MutanBEST kit. The mutation was confirmed by DNA sequencing. The *pENTR-MYC2^T328A^* was then combined with binary vector pGWB17 (35S promoter, C-4myc) [43] to generate *35S_pro_:MYC2^T328A^-4myc* construct. Similarly, we generated *35S_pro_:MYC2^S330A^-4myc*, *35S_pro_:MYC2^S334A^-4myc*, *35S_pro_:MYC2^T336A^-4myc* constructs. All primers used for DNA construct generation are listed in Table S1.

The above constructs were then transformed into *Agrobacterium tumefaciens* strain GV3101 (pMP90), which was used for transformation of Arabidopsis plants by vacuum infiltration [44].

### Transient Expression Assay in *N. benthamiana* Leaves

The transient expression assays were performed in *N. benthamiana* leaves as previously described [1], [45]. The *ORA59* promoter was amplified cloned into pENTR using the pENTR Directional TOPO cloning kit (Invirogen). To generate *ORA59* promoter with mutations, site-directed mutagenesis was used to delete the CACGTG motif in the P1 region of the *ORA59* promoter (Figure 2A) using the TaKaRa MutanBEST kit. Similarly, the *LOX2* promoter was amplified and cloned into pENTR vector. Then various promoter versions were fused with the luciferase reporter gene LUC through the Gateway reactions into the plant binary vector pGWB35 [43] to generate the reporter constructs *ORA59_pro_:LUC*, *ORA59_mpro_:LUC*, *LOX2_pro_:LUC*. The *MYC2* and *MYC2^ΔDE^* effector constructs were the above-described *35S_pro_:MYC2-GFP* (*35S_pro_:MYC2*), and *35S_pro_:MYC2^ΔDE^*-GFP (*35S_pro_:MYC2^ΔDE^*). We used a low-light cooled CCD imaging apparatus (NightOWL II LB983 with indigo software) to capture the LUC image and to count luminescence intensity. The leaves were sprayed with 100 mM luciferin and were placed in darkness for 3 min before luminescence detection.

### Transactivation Activity Assay in Yeast

Full-length coding sequence and TAD truncation of MYC2 were amplified with listed primers (see Table S1 online). Enzyme-digested PCR products were cloned into the NdeI and PstI sites of the vector pGBKT7. The resulting constructs were then transformed into the yeast strain *Saccharomyces cerevisiae* AH109. The MATCHMAKER GAL4-based Two-Hybrid System 3 (Clontech) was used for the transactivation activity assay. Each yeast liquid culture was serially diluted to OD600 = 0.6, and 5 µl of each dilution was inoculated onto SD/-Ade/-His/-Trp synthetic dropout medium. The expressed proteins in yeast strains were analyzed by immunoblot experiments. Proteins fused with the GAL4 DNA binding domain were detected using anti-myc antibody.

### Gene Expression Analysis

For qRT-PCR analysis, seedling were harvested and frozen in liquid nitrogen for RNA extraction. RNA extraction and qRT-PCR analysis were performed as previously described [27]. Primers used to quantify gene expression levels are listed in Table S2.

### Protein Extraction and Immunoblot Assays

Details for protein extraction and immunoblot assays were described recently [27]. Antibodies and dilutions used in these experiments were as follows: anti-myc antibody (Abmart, 1∶2000), anti-GFP antibody (Abmart, 1∶1000). anti-ubiquitin antibody (Sigma, 1∶1000).

### Coimmunoprecipitation

*N. benthamiana* leaves that transiently expressing *MYC2-4myc* or *MYC2-4myc-15* seedlings were homogenized with ice-cold extraction buffer (50 mM Tris-HCl, pH 7.5, 150 mM NaCl, 0.1% Triton X-100, 0.2% Nonidet P-40, 0.6 mM PMSF, and 20 µM MG132 with Roche protease inhibitor cocktail). After protein extraction, 20 µL protein G plus agarose (Santa Cruz) was added to the 2-mg extracts to reduce nonspecific immunoglobulin binding. After 1 h of incubation, the supernatant was transferred to a new tube. myc antibody-bound agarose beads (Santa Cruz) were then added to each reaction for 4 h at 4°C. The precipitated samples were washed at least four times with the extraction buffer and then eluted by adding 1× SDS protein loading buffer with boiling for 5 min.

A phosphor-portein enrichment kit (Clontech) was used to column-purify phospho-proteins according to the manufacturer's protocol.

### ChIP–PCR Assay

ChIP was performed as previously described [46]. 1.5 gram of 10-d-old *35S_pro_:MYC2-GFP* seedlings were used for ChIP experiments. GFP antibody (Abcam) was used to immunoprecipitate the protein-DNA complex. Chromatin precipitated without antibody was used as negative control, while the isolated chromatin before precipitation was used as input control. The enrichment of DNA fragments was determined by semiquantitative PCR. Primers used for ChIP-PCR were listed in Table S2.

### Electrophoretic Mobility Shift Assay

Recombinant MYC2 protein in *Escheichia coli* (*E. coli*) used in this assay was previously described [1]. Oligonucleotide probes were synthesized and labeled with biotin at the 3′ end (Invitrogen). EMSA was performed using a Lightshift Chemiluminescent EMSA Kit (Thermo Scientific). Briefly, biotin-labeled probes were incubated in 1× binding buffer, 2.5% glycerol, 50 mM KCl, 5 mM MgCl_2_ and 10 mM EDTA with or without proteins at room temperature for 20 min. For nonlabeled probe competition, nonlabeled probe was added to the reactions. The probe sequences used in MESA were listed in Table S3.

### Identification of MYC2 Phosphorylation Site

The anti-myc immunoprecitipitates from *MYC2-4myc-15* transgenic plants were resolved on SDS-PAGE and visualized by silver staining. A protein band of approximately 70 KD was cut from the gel and digested with trypsin overnight at 37°C. Peptides were extracted sequentially with 5% formic acid (FA)/50% acetonitrile and 0.1% FA/75% acetonitrile, dried under vacuum, then resuspended in 0.1% FA. LC-MS/MS and data analysis were performed as described previously [47] except that the peptide sample was loaded directly on an analytical reverse-phase column.

### Plant Infection

Grow *Botrytis cinerea* on MEA medium (2% malt extract, 2% glucose, 0.1% peptone, 2% agar) in Petri dishes for 14 days at 24°C with 12 h photoperiod before collection of spores. Spore inoculums were prepared by harvesting spores in water, filtration though nylon mesh to remove hyphae and suspension in potato dextrose broth to a concentration of 10^5^ spores/ml. Detached rosette leaves of 28-d-old plants were placed in Petri dishes containing 0.8% agar, with the petiole embedded in medium. Each leaf was inoculated with a single 5 µl droplet of *B. cinerea* inoculums. Trays were covered with lids and kept under the same conditions as for plant growth. Photographs were taken after 3 days and mean lesion sizes of 20 leaves from 20 plants of various genotypes were compared using a Student's t-test assuming equal variance.

## Supporting Information

Figure S1 Generation of Transgenic Plants Containing *35S_pro_:MYC2-GUS*, *35S_pro_:MYC2-GFP* or *35S_pro_:MYC2-4myc* in the Genetic Background of *myc2-2*. (A) Transgene expression levels in the indicated plants revealed by qRT-PCR analysis. Total RNA was extracted from 7-day-old seedlings for qRT-PCR analysis. qRT-PCR amplifications were normalized to the expression of *ACTIN7*. Values are mean ± SD of three replicates. (B) Ten-day-old seedlings of WT, *myc2-2* and *MYC2* overexpression lines were grown on 1/2 MS medium without or with 20 µM JA.
(PDF)

Figure S2 Time-Course Expression of *ORA59* in Response to MeJA Treatment. Seven-day-old WT and *myc2-2* seedlings were treated with 100 µM MeJA for indicated times before total RNAs were extracted for qRT-PCR assays. Values are mean ± SD of three replicates. The experiments were repeated three times with similar results.
(PDF)

Figure S3 CHX but not MG132 Induces the Transcription of *MYC2*. Seven-day-old seedlings of *MYC2-GUS-18*, *MYC2-GFP-12* and *MYC2-4myc-15* were treated with (+) or without (−) 100 µM CHX for 2 h or 50 µM MG132 for 6 h. Subsequently, the expression of *MYC2* was analyzed using qRT-PCR. qRT-PCR amplifications were normalized to the expression of *ACTIN7*. Values are mean ± SD of three replicates.
(PDF)

Figure S4 Effect of CHX or MG132 on MeJA-induced Transcription of *MYC2*. (A) Seven-day-old seedlings of *MYC2-GFP-12* and *MYC2-4myc-15* were treated with 100 µM MeJA and/or 100 µM CHX for 6 h and the expression of *MYC2* was quantified with qRT-PCR. qRT-PCR amplifications were normalized to the expression of *ACTIN7*. Values are mean ± SD of three replicates. (B) Seven-day-old seedlings of *MYC2-GFP-12* and *MYC2-4myc-15* plants were treated treated with 100 µM MeJA and/or 50 µM MG132 for indicated times and the expression of *MYC2* was analyzed using qRT-PCR. qRT-PCR amplifications were normalized to the expression of *ACTIN7*. Values are mean ± SD of three replicates.
(PDF)

Figure S5 Immunoblot Analysis of MYC2 and MYC2ΔTAD Proteins in Yeast Shown in Figure 5B. Total proteins were extracted from the yeast cells expressing BD-MYC2 and BD-MYC2ΔTAD, loaded on SDS-PAGE, and detected with anti-myc antibody.
(PDF)

Figure S6 Generation of Transgenic Plants Containing *35S_pro_:MYC2^ΔDE^-GFP* or *35S_pro_:MYC2-GFP* in the Genetic Background of *myc2-2*. Transgene expression levels in the indicated plants revealed by RT-PCR analysis. Total RNA was extracted from seven-day-old seedlings for RT-PCR analysis. Expression of *ACTIN7* was used as an internal control.
(PDF)

Figure S7 MYC2^ΔDE^ Still Bind to the Promoter of *ORA59*. Enrichment of the DNA fragment (P1) shown in Figure 2A following ChIP using anti-GFP antibody. Chromatin of transgenic plant *MYC2* ^ΔDE^ *-GFP-6* was immuno-precipitated with an anti-GFP antibody, and the presence of the indicated DNA in the immune complex was determined by RT-PCR. The *ACTIN2* promoter fragment was used as a negative control.
(PDF)

Figure S8 Generation of Transgenic Plants Containing Indicated Point Mutations of the *MYC2* Gene in the Genetic Background of *myc2-2*. Transgene expression levels in the indicated plants revealed by qRT-PCR analysis. Total RNA was extracted from seven-day-old seedlings for qRT-PCR analysis. qRT-PCR amplifications were normalized to the expression of *ACTIN7*. Values are mean ± SD of three replicates.
(PDF)

Table S1 DNA primer pairs used for construct generation.
(PDF)

Table S2 DNA primer pairs used for qRT-PCR and ChIP-PCR assays.
(PDF)

Table S3 Oligonucleotide probes used in EMSA.
(PDF)
