# Supplementary figures and images for: Phosphorylation-Coupled Proteolysis of the Transcription Factor MYC2 Is Important for Jasmonate-Signaled Plant Immunity
Source: PLoS Genet. 2013 Apr 4;9(4):e1003422. doi: 10.1371/journal.pgen.1003422 (PMC3616909; doi:10.1371/journal.pgen.1003422)

A

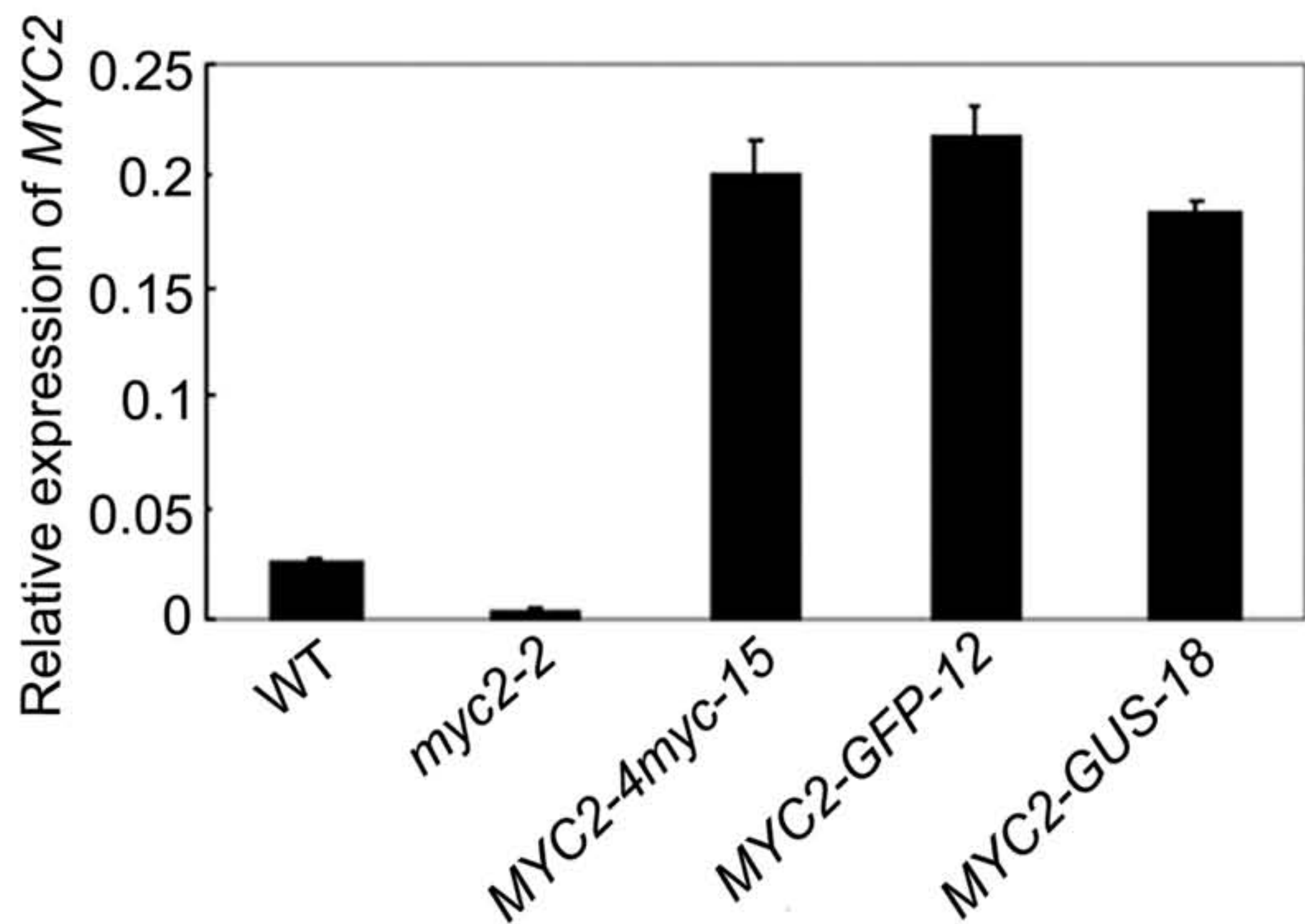

B

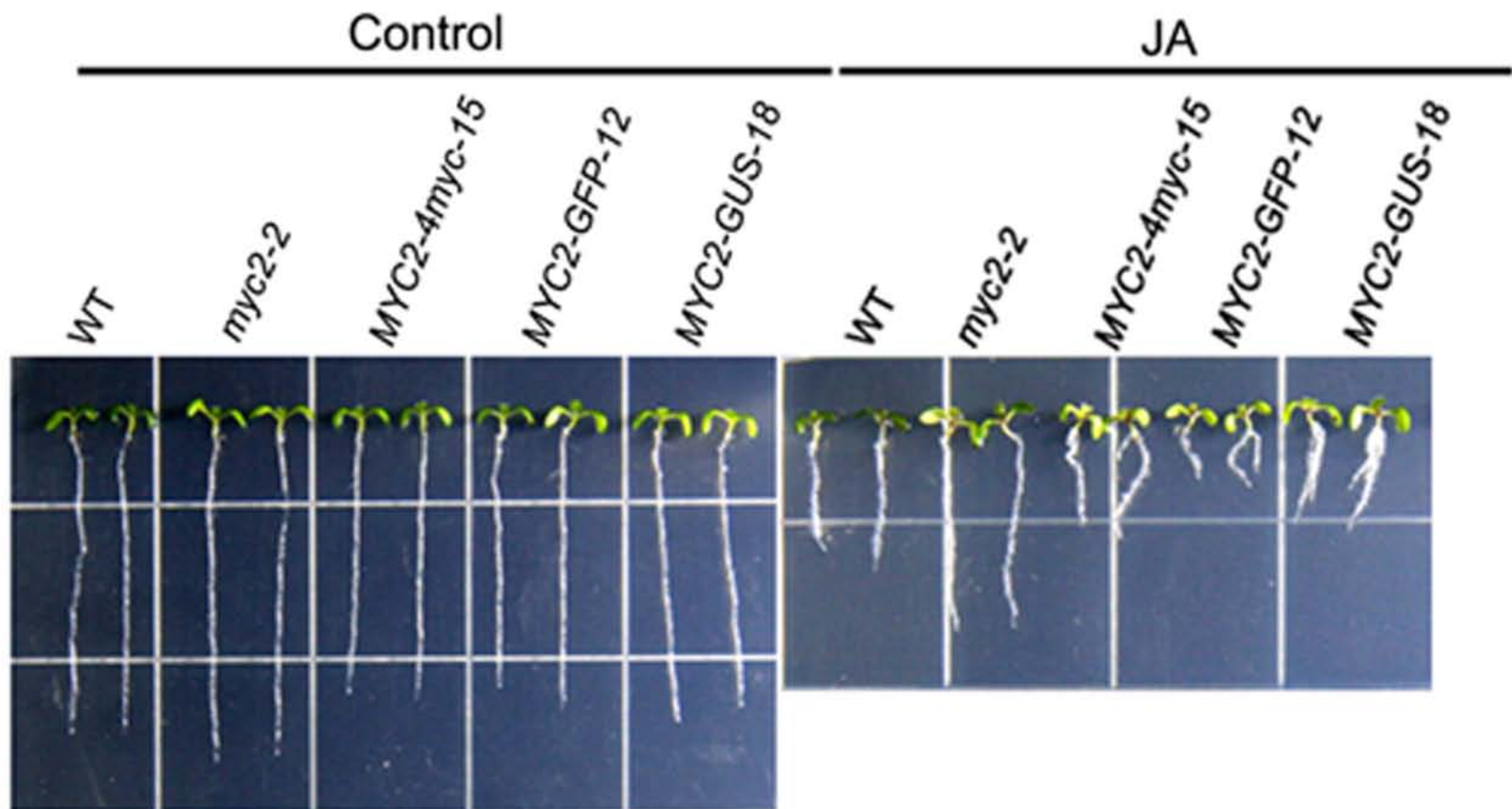

Supplement: Figure S1 — Generation of Transgenic Plants Containing 35Spro:MYC2-GUS, 35Spro:MYC2-GFP or 35Spro:MYC2-4myc in the Genetic Background of myc2-2. (A) Transgene expression levels in the indicated plants revealed by qRT-PCR analysis. Total RNA was extracted from 7-day-old seedlings for qRT-PCR analysis. qRT-PCR amplifications were normalized to the expression of ACTIN7. Values are mean ± SD of three replicates. (B) Ten-day-old seedlings of WT, myc2-2 and MYC2 overexpression lines were grown on 1/2 MS medium without or with 20 µM JA. (PDF) [file pgen.1003422.s001.pdf]

Zhai et al., Figure S2

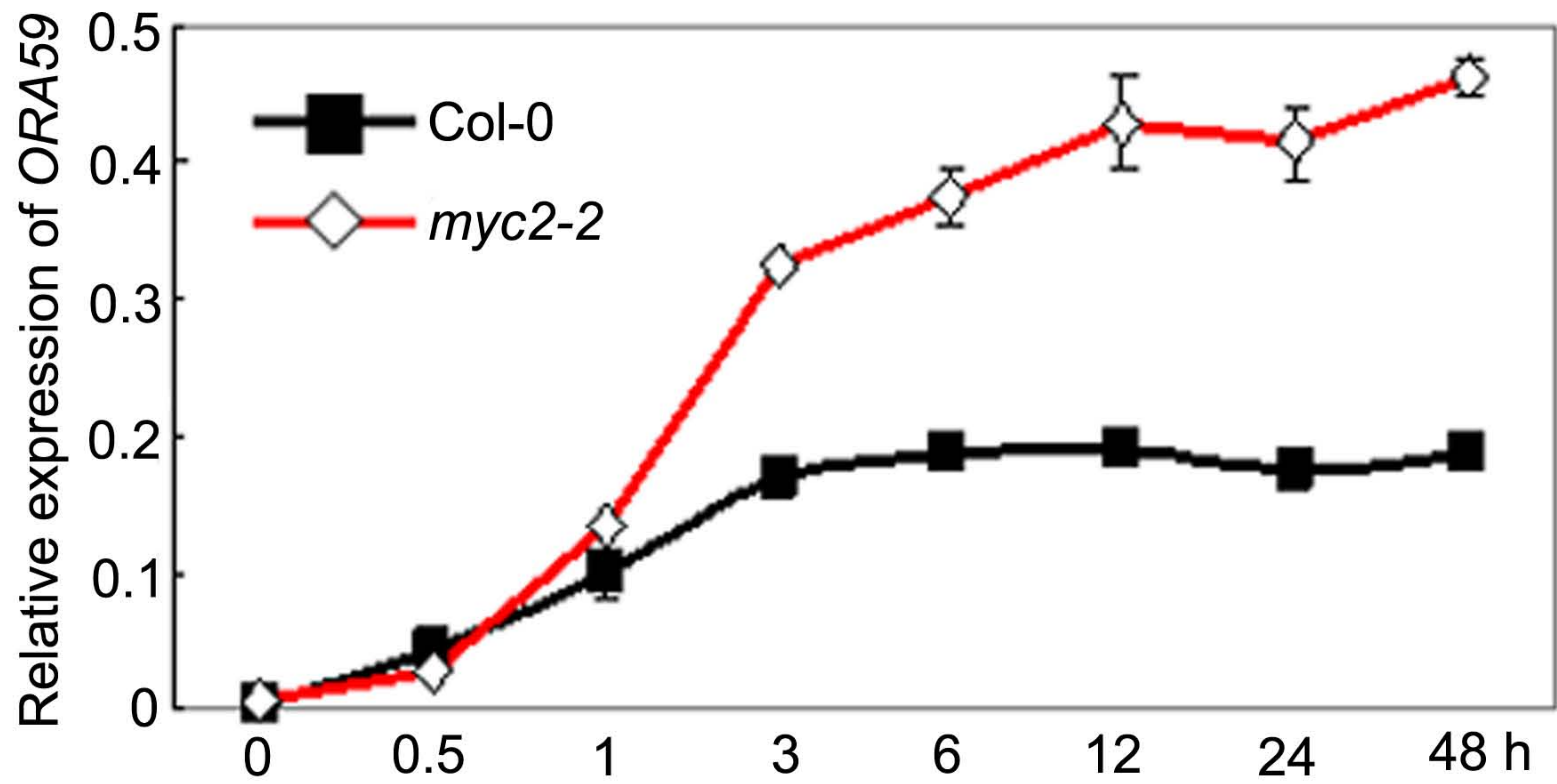

Supplement: Figure S2 — Time-Course Expression of ORA59 in Response to MeJA Treatment. Seven-day-old WT and myc2-2 seedlings were treated with 100 µM MeJA for indicated times before total RNAs were extracted for qRT-PCR assays. Values are mean ± SD of three replicates. The experiments were repeated three times with similar results. (PDF) [file pgen.1003422.s002.pdf]

Zhai et al., Figure S3

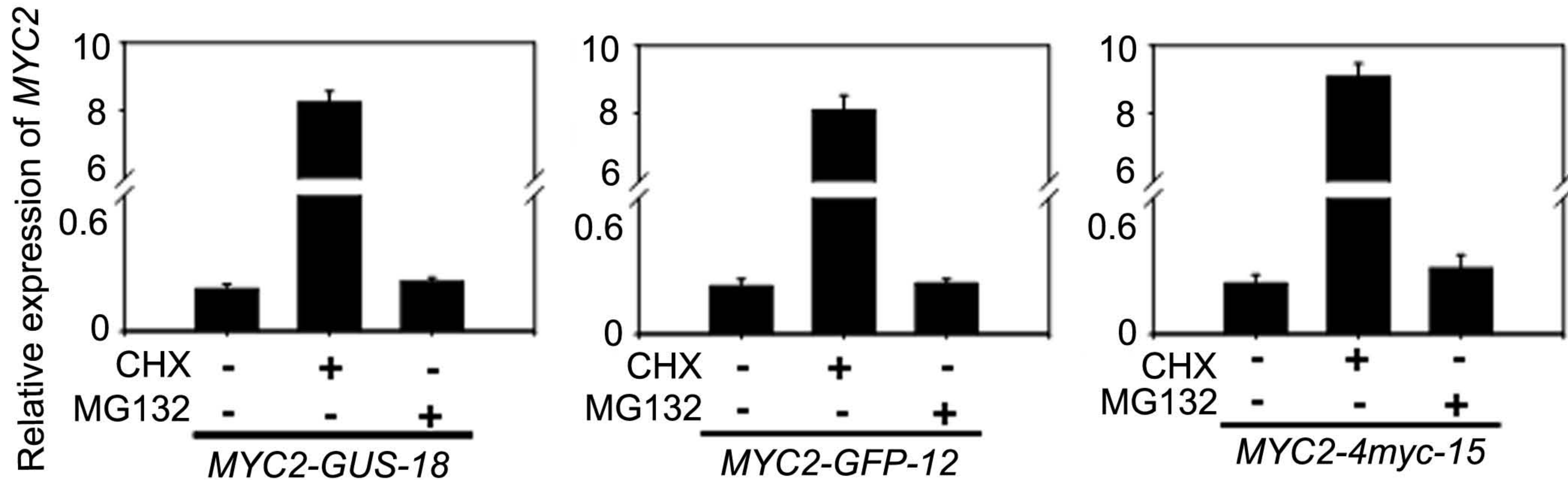

Supplement: Figure S3 — CHX but not MG132 Induces the Transcription of MYC2. Seven-day-old seedlings of MYC2-GUS-18, MYC2-GFP-12 and MYC2-4myc-15 were treated with (+) or without (−) 100 µM CHX for 2 h or 50 µM MG132 for 6 h. Subsequently, the expression of MYC2 was analyzed using qRT-PCR. qRT-PCR amplifications were normalized to the expression of ACTIN7. Values are mean ± SD of three replicates. (PDF) [file pgen.1003422.s003.pdf]

A

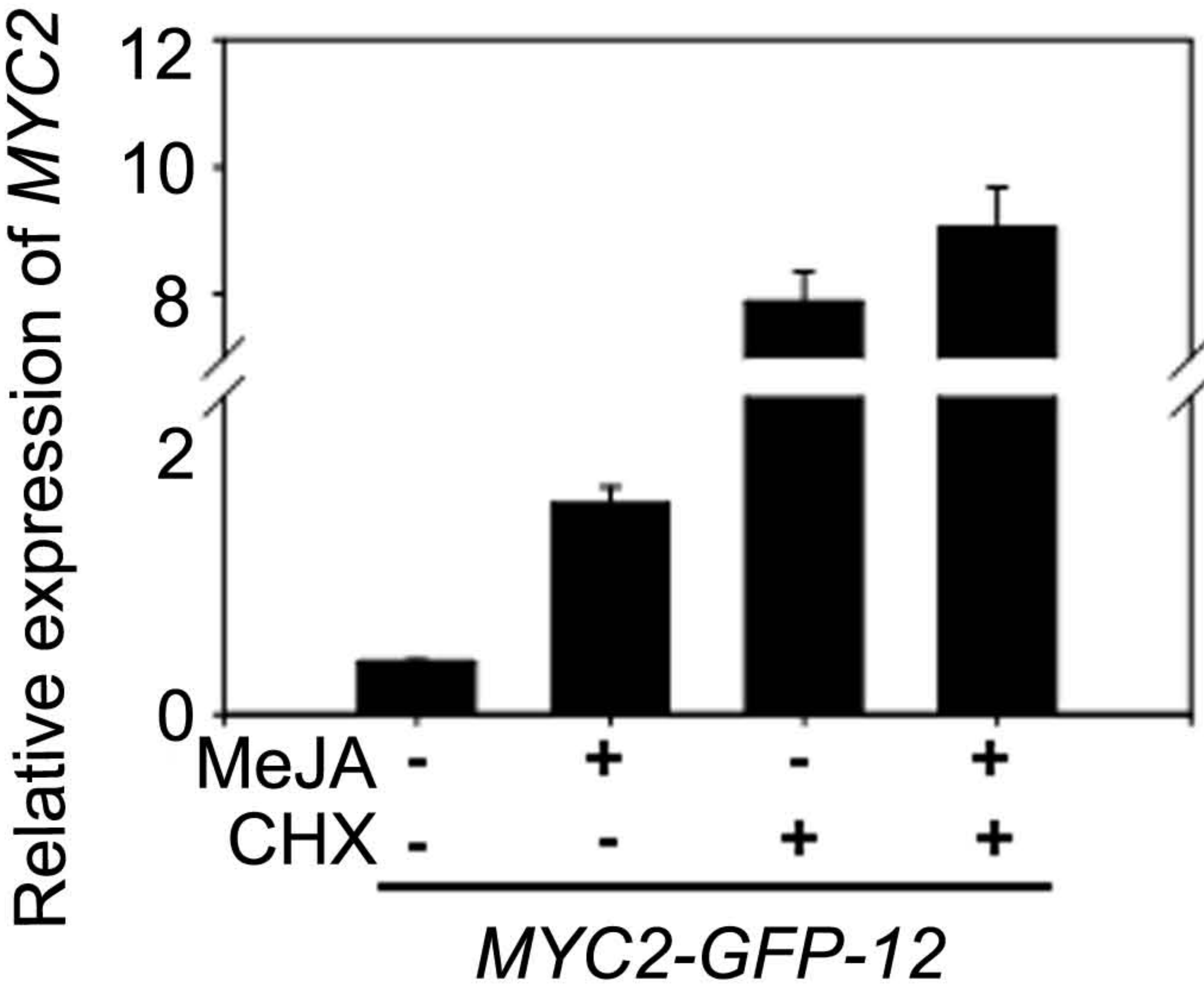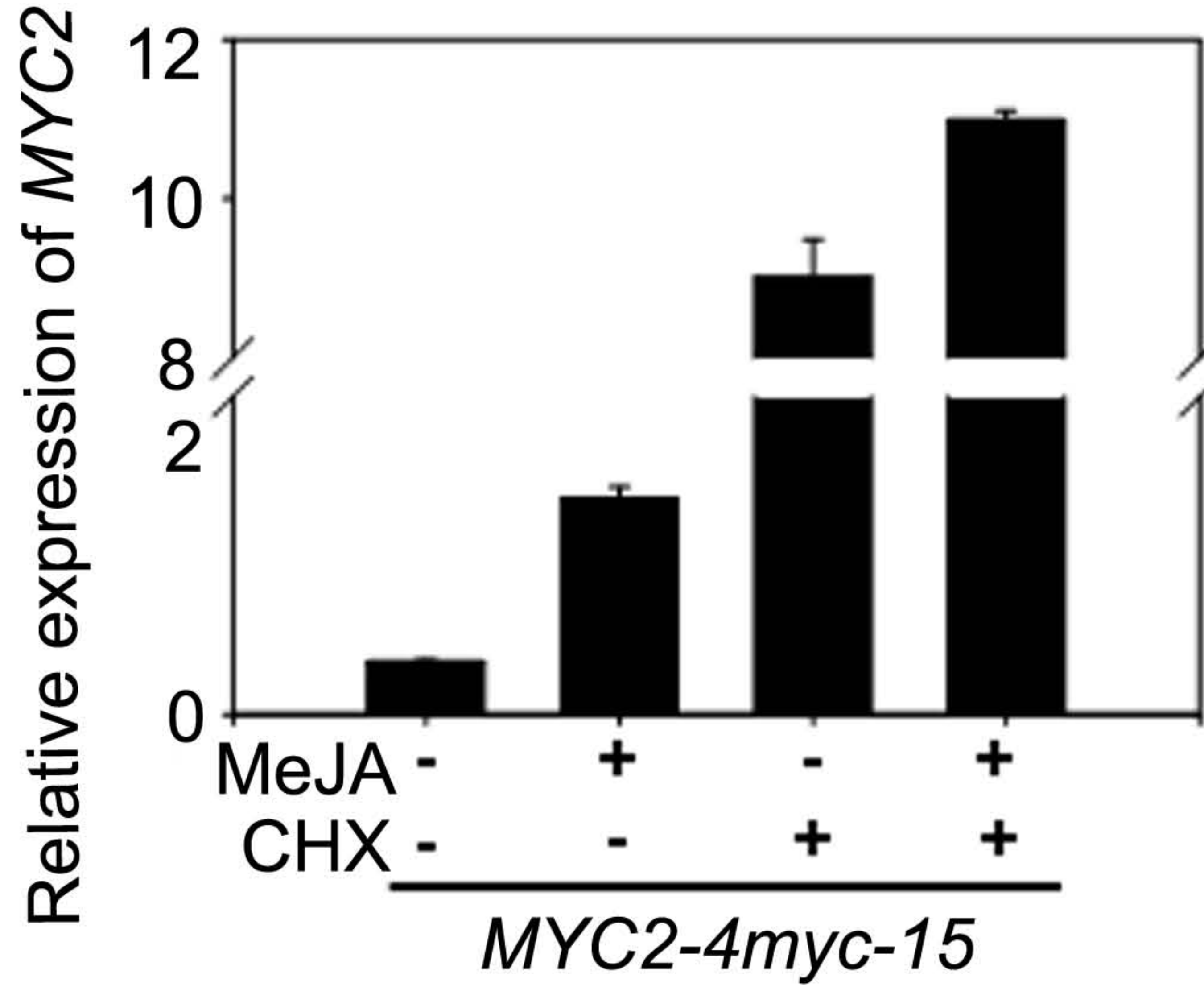

B

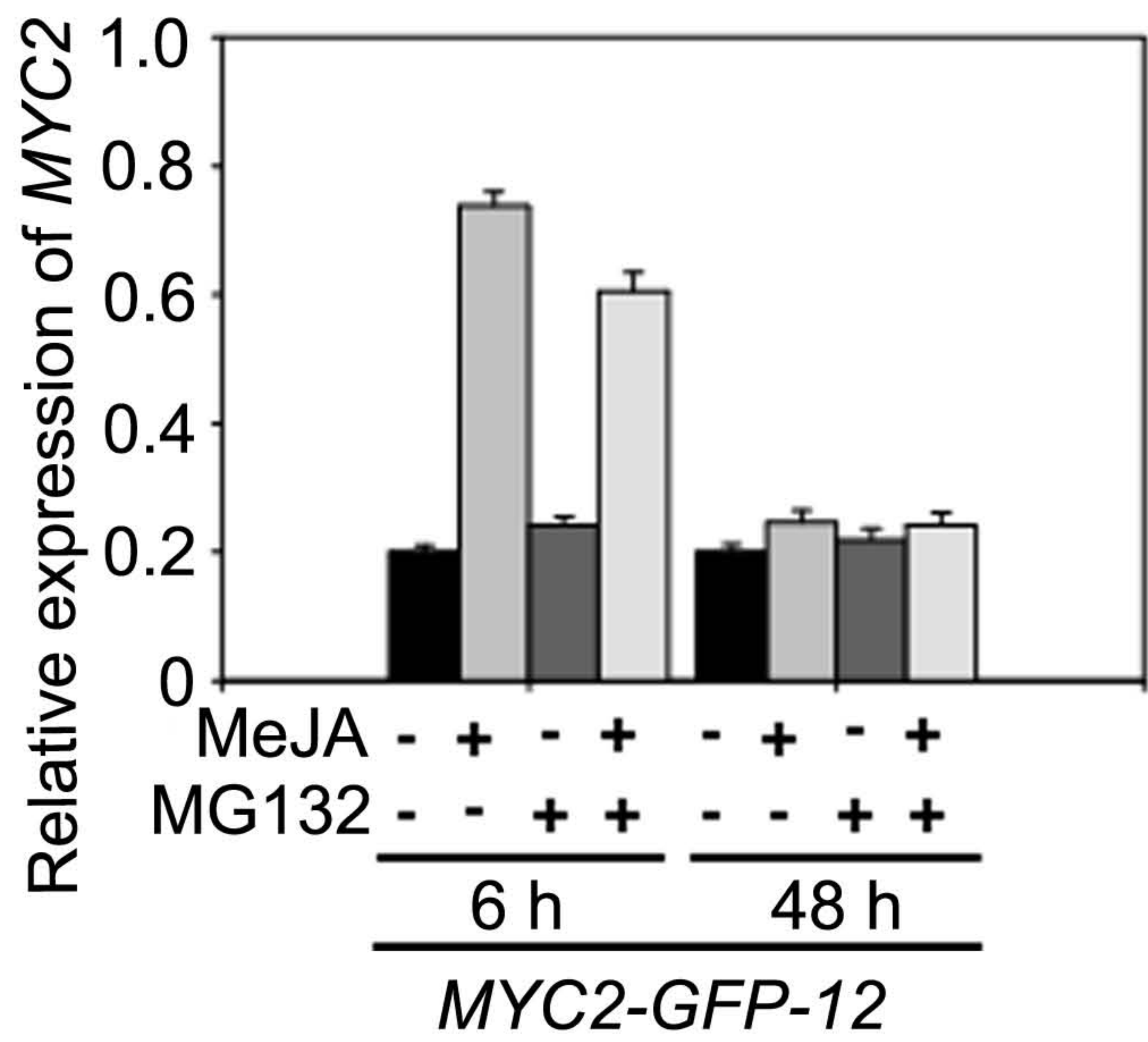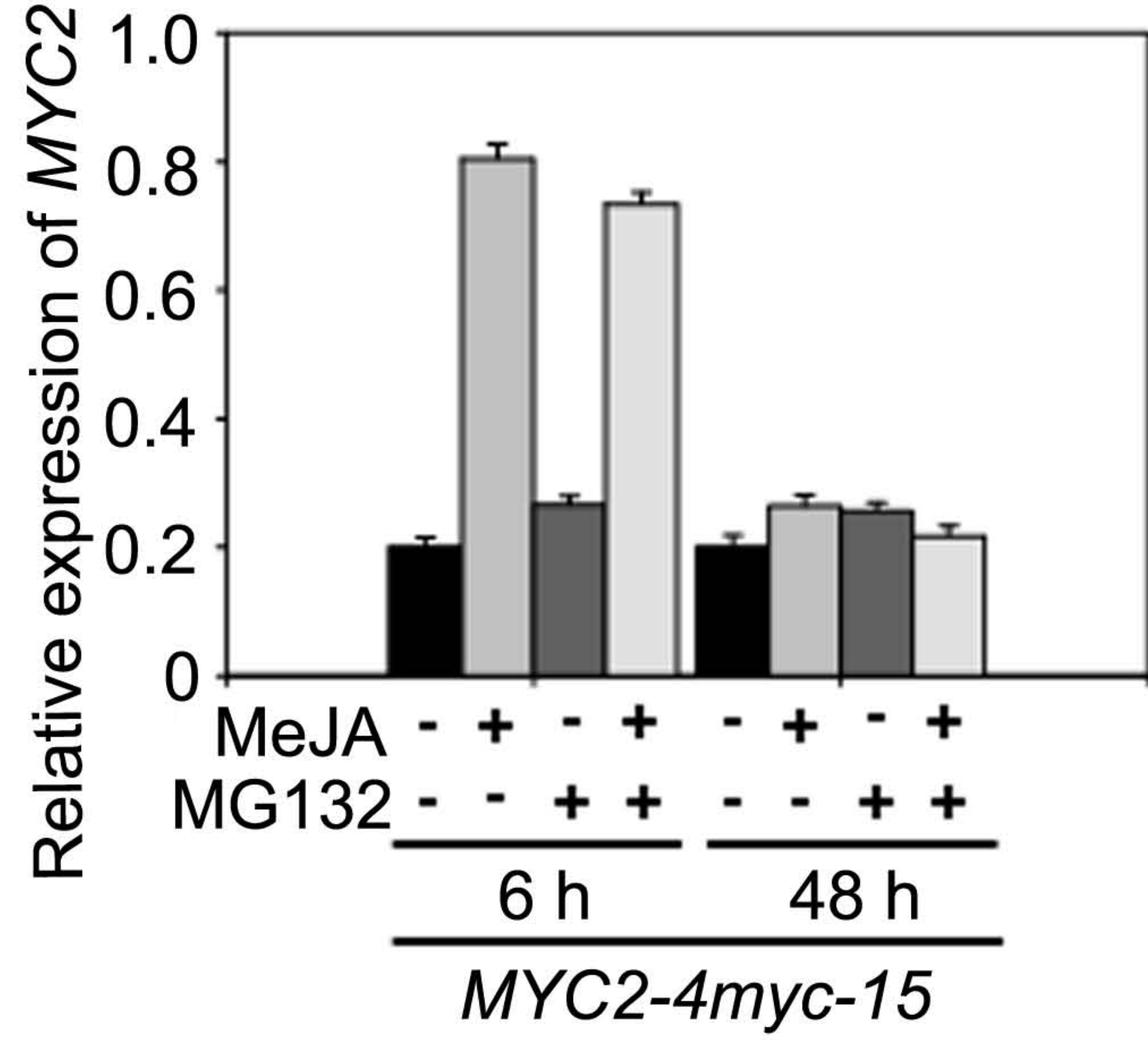

Supplement: Figure S4 — Effect of CHX or MG132 on MeJA-induced Transcription of MYC2. (A) Seven-day-old seedlings of MYC2-GFP-12 and MYC2-4myc-15 were treated with 100 µM MeJA and/or 100 µM CHX for 6 h and the expression of MYC2 was quantified with qRT-PCR. qRT-PCR amplifications were normalized to the expression of ACTIN7. Values are mean ± SD of three replicates. (B) Seven-day-old seedlings of MYC2-GFP-12 and MYC2-4myc-15 plants were treated treated with 100 µM MeJA and/or 50 µM MG132 for indicated times and the expression of MYC2 was analyzed using qRT-PCR. qRT-PCR amplifications were normalized to the expression of ACTIN7. Values are mean ± SD of three replicates. (PDF) [file pgen.1003422.s004.pdf]

# Zhai et al., Figure S5

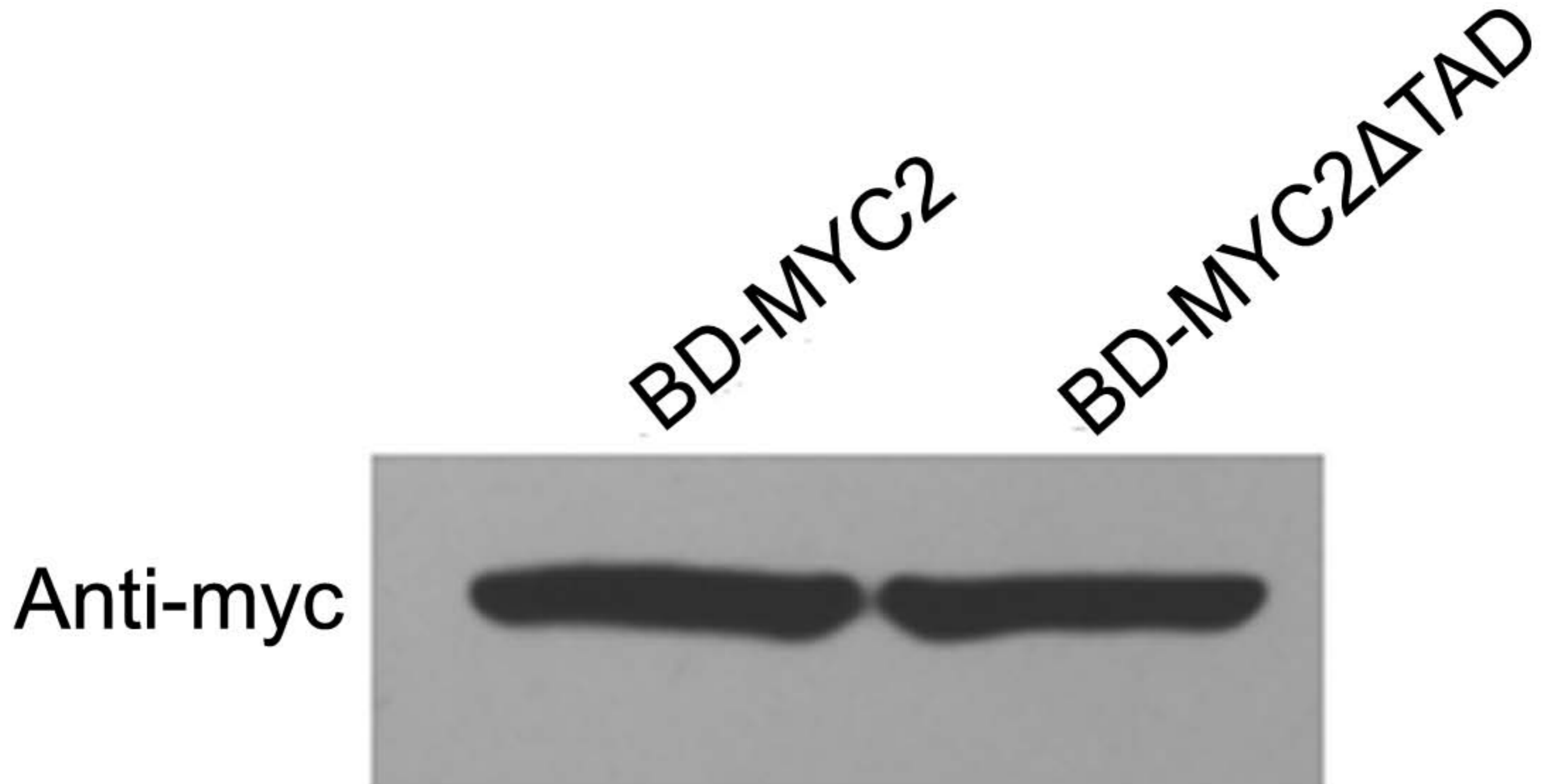

Supplement: Figure S5 — Immunoblot Analysis of MYC2 and MYC2ΔTAD Proteins in Yeast Shown in Figure 5B. Total proteins were extracted from the yeast cells expressing BD-MYC2 and BD-MYC2ΔTAD, loaded on SDS-PAGE, and detected with anti-myc antibody. (PDF) [file pgen.1003422.s005.pdf]

# Zhai et al., Figure S6

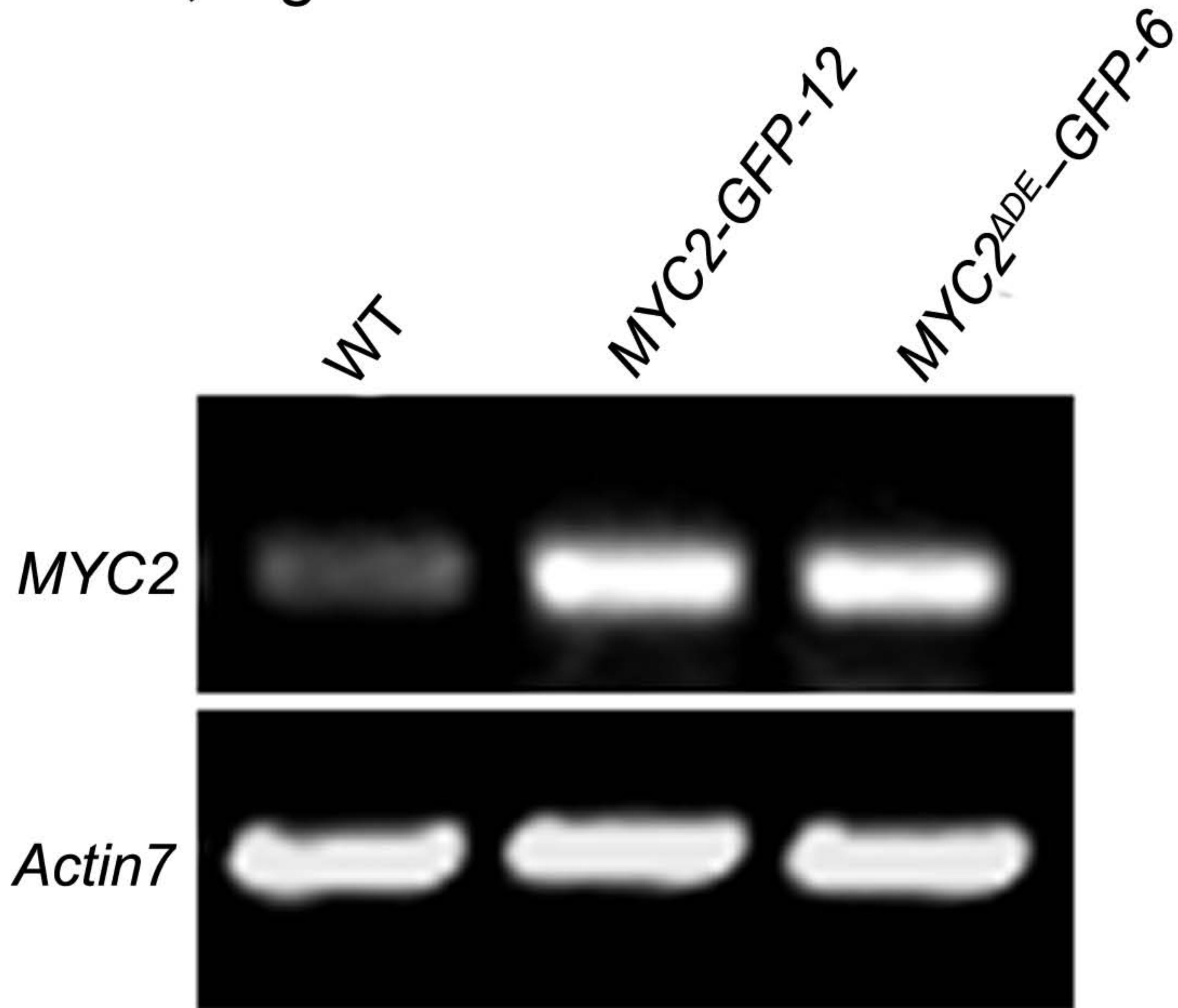

Supplement: Figure S6 — Generation of Transgenic Plants Containing 35Spro:MYC2ΔDE-GFP or 35Spro:MYC2-GFP in the Genetic Background of myc2-2. Transgene expression levels in the indicated plants revealed by RT-PCR analysis. Total RNA was extracted from seven-day-old seedlings for RT-PCR analysis. Expression of ACTIN7 was used as an internal control. (PDF) [file pgen.1003422.s006.pdf]

# Zhai et al., Figure S7

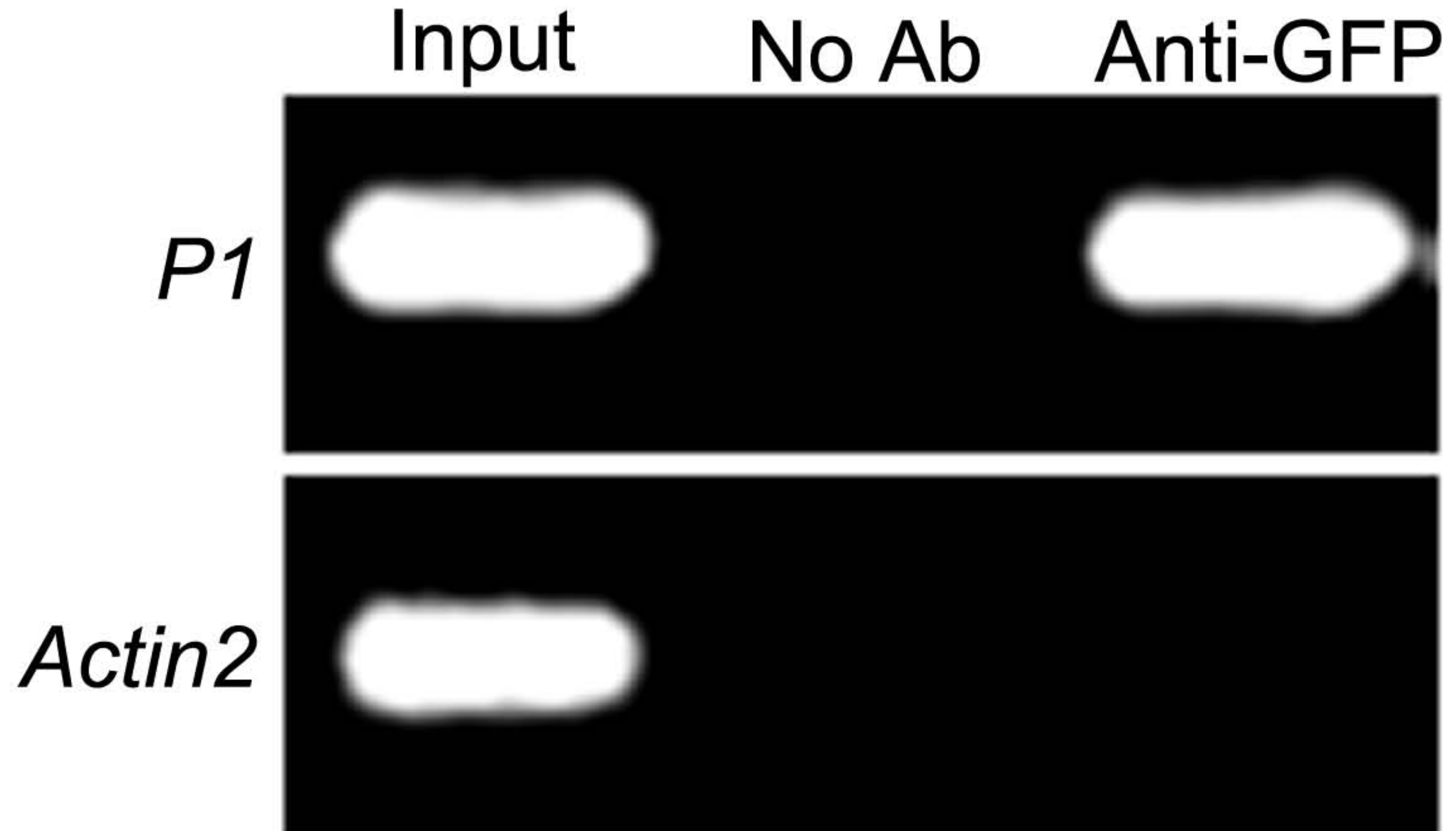

Supplement: Figure S7 — MYC2ΔDE Still Bind to the Promoter of ORA59. Enrichment of the DNA fragment (P1) shown in Figure 2A following ChIP using anti-GFP antibody. Chromatin of transgenic plant MYC2 ΔDE -GFP-6 was immuno-precipitated with an anti-GFP antibody, and the presence of the indicated DNA in the immune complex was determined by RT-PCR. The ACTIN2 promoter fragment was used as a negative control. (PDF) [file pgen.1003422.s007.pdf]

# Zhai et al., Figure S8

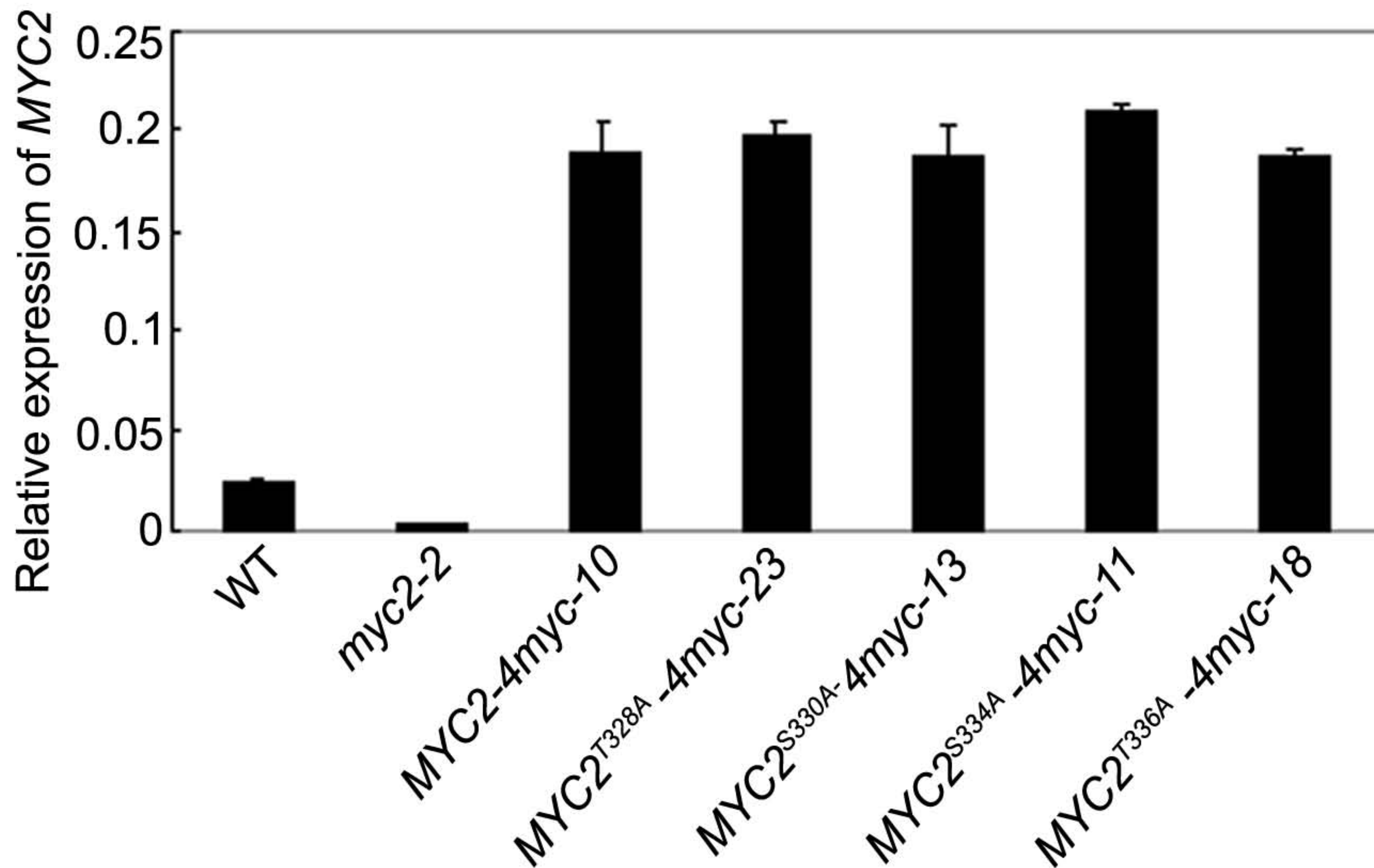

Supplement: Figure S8 — Generation of Transgenic Plants Containing Indicated Point Mutations of the MYC2 Gene in the Genetic Background of myc2-2. Transgene expression levels in the indicated plants revealed by qRT-PCR analysis. Total RNA was extracted from seven-day-old seedlings for qRT-PCR analysis. qRT-PCR amplifications were normalized to the expression of ACTIN7. Values are mean ± SD of three replicates. (PDF) [file pgen.1003422.s008.pdf]
