# Supplementary material for: Phosphorylation-Coupled Proteolysis of the Transcription Factor MYC2 Is Important for Jasmonate-Signaled Plant Immunity
Source: PLoS Genet. 2013 Apr 4;9(4):e1003422. doi: 10.1371/journal.pgen.1003422 (PMC3616909; doi:10.1371/journal.pgen.1003422)
Supplement: Table S1 — DNA primer pairs used for construct generation. (PDF) [file pgen.1003422.s009.pdf]

Zhai et al., Table S1

Table S1. DNA Primer Pairs Used for Construct Generation.

| Name | Primer sequence (5'-3') |
|---|---|
| TOPO-MYC2-F | CACCATGACTGATTACCGGCTACA |
| TOPO-MYC2-R | ACCGATTTTTGAAATCAAACTTGC |
| TOPO-ORA59-F | CACCATGGAATATCAAACTAACTTC |
| TOPO-ORA59-R | CAAGAACATGATCTCATAAGCT |
| TOPO-ORA59$_{pro}$-F | CACCAACGTATAAGTACTTCTACTG |
| TOPO-ORA59$_{pro}$-R | CTCTCCACTTAAGAAGTTAGTTTG |
| TOPO-LOX2$_{pro}$-F | CACCAACAAAGTAGCCCTATAGGGT |
| TOPO-LOX2$_{pro}$-R | CGACAAGGACTCTCTACAAT ACAT |
| MYC2-KpnI-F | GCGGGTACCATGACTGATTACCGGCTACAACC |
| MYC2-SpeI-R | CGCACTAGTACCGATTTTTGAAATCAAACTTGC |
| GUS-SalI-F | GCCGTCGACATGTTACGTCCTGTAGAAACCC |
| GUS-PstI-R | CTCCTGCAGTCATTGTTTGCCTCCCTGCTGC |
| ORA59$_{mpro}$-F | GAACATTTCACCCACTTCTGTG |
| ORA59$_{mpro}$-R | GGACAAGACCAGGTTGAGTGTA |
| MYC2$^{\Delta DE}$ -F | GTTGCTCCGTCGTGGTTTTTCTTG |
| MYC2$^{\Delta DE}$ -R | CAAGAAAAACCACGACGGAGCAAC |
| MYC2$^{T328A}$ -F | AACCCGAATCTGGATCCGGCT |
| MYC2$^{T328A}$ -R | TTCGGTTATTGTGCTTGAGCT |
| MYC2$^{S330A}$ -F | CGACTCCGGCTCCGGTTCATT |
| MYC2$^{S330A}$ -R | GATCCAGATTCGGGTTTTCGG |
| MYC2$^{S334A}$ -F | ACTCCGAGTCCGGTTCATGCT |
| MYC2$^{S334A}$ -R | CGGATCCAGATTCGGGTTTTC |
| MYC2$^{T336A}$ -F | GGTTCATTCTCAGGCCCAGAA |
| MYC2$^{T336A}$ -R | GGACTCGGAGTCGGATCCAGA |
| MYC2ΔTAD-F | TTGATCTCCGGTACGGGTAACGCG |
| MYC2ΔTAD-R | CGCGTTACCCGTACCGGAGATCAA |
| BD-MYC2-F | CCATTGCATATGACTGATTACCGGCTACA |
| BD-MYC2-R | AAGCTGCAGACCGATTTTTGAAATCAAAC |
