# Supplementary material for: Phosphorylation-Coupled Proteolysis of the Transcription Factor MYC2 Is Important for Jasmonate-Signaled Plant Immunity
Source: PLoS Genet. 2013 Apr 4;9(4):e1003422. doi: 10.1371/journal.pgen.1003422 (PMC3616909; doi:10.1371/journal.pgen.1003422)
Supplement: Table S2 — DNA primer pairs used for qRT-PCR and ChIP-PCR assays. (PDF) [file pgen.1003422.s010.pdf]

Zhai et al., Table S2

Table S2. DNA Primer Pairs Used for qRT-PCR and ChIP-PCR Assays.

Name	Primer sequence (5'-3')
LOX2-Q-F	GCAACGCTACGGGGGAGAG
LOX2-Q-R	ACTGGGGCATCAAACTGGAGAAT
PDF1.2-Q-F	CGCACCGGCAATGGTGGAAG
PDF1.2-Q-R	CACACGATTTAGCACCAAAG
ORA59-Q-F	CACACTCTTATCCCTTTCCTCGTT
ORA59-Q-R	AATAGGAGGAGGAGGAAGAAGGAG
MYC2-Q-F	CGACGGCGGAGCTGGAGATTTAT
MYC2-Q-R	GATTCGGGTTTTCGGTTATTGTGC
ACTIN 7-Q-F	CCATTCAGGCCGTTCTTTC
ACTIN 7-Q-F	CGTTCTGCGGTAGTGGTGA
ORA59-ChIP-F	GTACGTCATACACTCAACCTG
ORA59-ChIP-R	CAATTAGGCTGCCTCCGAATA
ACTIN2-ChIP-F	CGTTTCGCTTTCCTTAGTGTTAGCT
ACTIN2-ChIP-R	CACAACGCATGCTAAACAGATCTAG
