# Supplementary material for: Phosphorylation-Coupled Proteolysis of the Transcription Factor MYC2 Is Important for Jasmonate-Signaled Plant Immunity
Source: PLoS Genet. 2013 Apr 4;9(4):e1003422. doi: 10.1371/journal.pgen.1003422 (PMC3616909; doi:10.1371/journal.pgen.1003422)
Supplement: Table S3 — Oligonucleotide probes used in EMSA. (PDF) [file pgen.1003422.s011.pdf]

Zhai et al., Table S3

Table S3. Oligonucleotide Probes Used in EMSA.

Name	Sequence (5’-3’)
ORA59-F	TCAACCTGGTCTTGTCCcacgtgGAACATTTCACCCACTTCTG
ORA59-R	CAGAAGTGGGTGAAATGTTCcacgtgGGACAAGACCAGGTTGA
ORA59mu-F	CAGAAGTGGGTGAAATGTTCaaaaaaGGACAAGACCAGGTTGA
ORA59mu-R	TCAACCTGGTCTTGTCCttttttGAACATTTCACCCACTTCTG
